# The Well-Being Benefits of Person-Culture Match Are Contingent on Basic Personality Traits

**DOI:** 10.1177/0956797620951115

**Published:** 2020-09-14

**Authors:** Jochen E. Gebauer, Jennifer Eck, Theresa M. Entringer, Wiebke Bleidorn, Peter J. Rentfrow, Jeff Potter, Samuel D. Gosling

**Affiliations:** 1 Department of Psychology, University of Mannheim; 2 Department of Psychology, University of Copenhagen; 3 German Institute for Economic Research, Berlin, Germany; 4 Department of Psychology, University of California, Davis; 5 Department of Psychology, University of Cambridge; 6 Atof Inc., Cambridge, Massachusetts; 7 Department of Psychology, University of Texas at Austin; 8 Department of Psychology, University of Melbourne

**Keywords:** person-culture match, culture, basic personality traits, Big Two, Big Five, open data, open materials

## Abstract

People enjoy well-being benefits if their personal characteristics match those of their culture. This *person-culture match effect* is integral to many psychological theories and—as a driver of migration—carries much societal relevance. But do people differ in the degree to which person-culture match confers well-being benefits? In the first-ever empirical test of that question, we examined whether the person-culture match effect is moderated by basic personality traits—the Big Two and Big Five. We relied on self-reports from 2,672,820 people across 102 countries and informant reports from 850,877 people across 61 countries. Communion, agreeableness, and neuroticism exacerbated the person-culture match effect, whereas agency, openness, extraversion, and conscientiousness diminished it. People who possessed low levels of communion coupled with high levels of agency evidenced no well-being benefits from person-culture match, and people who possessed low levels of agreeableness and neuroticism coupled with high levels of openness, extraversion, and conscientiousness even evidenced well-being costs. Those results have implications for theories building on the person-culture match effect, illuminate the mechanisms driving that effect, and help explain failures to replicate it.

The match between people’s personal characteristics and the characteristics of their culture is a wellspring of well-being (Diener, Oishi, & Tay, 2018). In his seminal work on this *person-culture match effect*, Rosenberg (1965) assumed that people enjoy self-esteem benefits if their own religion matches the religion of their sociocultural context. Diener, Tay, and Myers (2011) elaborated on why person-culture match confers well-being benefits: People who match are by definition similar to the people around them, are therefore liked more by those people, and receive more social support from them as a result. Social support, in turn, confers well-being benefits (Stavrova, Fetchenhauer, & Schlösser, 2013). Large-scale investigations have provided support for the person-culture match effect. For example, religious people are better adjusted in religious countries (Gebauer, Sedikides, & Neberich, 2012), wealthy people are happier in wealthy countries (Tay, Morrison, & Diener, 2014), and virtuous people are more satisfied with their lives in civically virtuous countries (Stavrova, Schlösser, & Fetchenhauer, 2013).

The person-culture match effect is integral to many psychological theories. For instance, this effect is a building block of numerous self-esteem theories, including terror management theory (Pyszczynski, Greenberg, Solomon, Arndt, & Schimel, 2004) and the self-enhancement tactician model (Sedikides, Gaertner, & Cai, 2015). The person-culture match effect also carries implications for pressing societal challenges. It is, for example, a key mechanism in contemporary explanations of immigration (pursuing the benefits of high match; Motyl, Iyer, Oishi, Trawalter, & Nosek, 2014) and emigration (escaping the costs of low match; Jokela, Elovainio, Kivimäki, & Keltikangas-Järvinen, 2008).

In the domain of person-culture match, the most timely, unanswered set of questions is perhaps this (cf. Kristof-Brown & Guay, 2011, p. 40): Do individuals differ in the degree to which person-culture match confers well-being benefits? And, if so, might some people benefit not at all from person-culture match? Or might some people even benefit from mismatch? Those questions are timely because they carry far-reaching implications (mentioned briefly here and elaborated on in the Discussion section). First, many psychological theories build on the person-culture match effect (reviewed by Gebauer et al., 2015). If person-culture match conferred benefits to some people only, those theories would benefit from incorporating individual differences. Second, the processes that drive the person-culture match effect are insufficiently understood (Diener et al., 2011). Individual-difference moderators would help illuminate those processes. Finally, the evidence for the person-culture match effect is inconsistent (Bleidorn et al., 2016). The existence of heretofore “hidden” individual-difference moderators may explain those inconsistencies.

The present research is a foray into individual-difference moderators of the person-culture match effect. As moderators, we focused on two basic personality taxonomies—the Big Two (communion and agency; Paulhus & John, 1998) and the Big Five (agreeableness, conscientiousness, openness to experience, extraversion, and neuroticism; John, Naumann, & Soto, 2008). We chose those taxonomies for two reasons. First, both are widely considered exhaustive at their level of abstraction (John et al., 2008; Paulhus & John, 1998) and are therefore ideal for broad investigations such as ours. Second, and more important, we had strong theoretical reasons to expect that the person-culture match effect would be contingent on the Big Two and on the Big Five.

## Theoretical Predictions

Regarding the Big Two, communion motivates assimilation to sociocultural norms (Gebauer, Leary, & Neberich, 2012; Gebauer, Paulhus, & Neberich, 2013; Gebauer, Sedikides, Lüdtke, & Neberich, 2014) and, thus, should elicit strivings for high person-culture match. Achieving personal strivings increases well-being (Emmons, 1986), so communal people in particular should benefit from person-culture match (i.e., more than the average person, who also benefits but solely because of consequences that apply to everyone, such as higher social support; Diener et al., 2011). Agency motivates contrast from sociocultural norms and, thus, should elicit strivings for low person-culture match (Gebauer, Leary, & Neberich, 2012; Gebauer et al., 2013; Gebauer, Sedikides, et al., 2014). Failing to achieve personal strivings decreases well-being (Emmons, 1986), so agentic people in particular should benefit little from person-culture match (but they may still benefit somewhat because they, too, should benefit from consequences that apply to everyone, such as higher social support).

**Statement of Relevance**

People enjoy well-being benefits if their personal characteristics match those of their culture. For example, people tend to have higher self-esteem if their own degree of religiosity matches the degree of religiosity of their larger social context. In this research, we tested whether the well-being benefits derived from this so-called person-culture match differ as a function of an individual’s personality. In other words, we asked whether some individuals benefit from person-culture match, whereas others do not, and whether still others might even benefit from mismatch. To address the research question, we relied on self-reports of personality from more than 2,500,000 people across 102 countries. As predicted, we found that some personality configurations were associated with stronger benefits from person-culture match, whereas others were associated with smaller benefits. There was even one personality configuration that seemed to fare better when people were mismatched with their culture. These findings are important because person-culture match carries societal relevance and is thought to be a driver of migration for people around the globe.

Regarding the Big Five, agreeableness and conscientiousness motivate assimilation to sociocultural norms (Ashton & Lee, 2019; Entringer et al., in press; Gebauer, Bleidorn, et al., 2014) and, thus, should elicit strivings for high person-culture match. Achieving personal strivings increases well-being (Emmons, 1986), so people high in agreeableness and conscientiousness in particular should benefit from person-culture match. Openness to experience motivates contrast from sociocultural norms and, thus, should elicit strivings for low person-culture match (Ashton & Lee, 2019; Entringer et al., in press; Gebauer, Bleidorn, et al., 2014). Failing to achieve personal strivings decreases well-being (Emmons, 1986), so people high in openness to experience in particular should benefit little from person-culture match (but they may still benefit somewhat because they, too, should benefit from consequences that apply to everyone, such as higher social support).

## Present Empirical Research

In our main study (reported here), we relied on data from 2,672,820 participants across 102 countries. The study used (a) *self-reports* as the reporting method (the most widely used method in the person-culture match literature; Diener et al., 2011), (b) *countries* as the units of culture (the most widely used units to define culture; Fulmer et al., 2010), (c) *religiosity* as the person-culture match domain (the classic match domain, in which person-culture match is probably best documented; Stavrova, Fetchenhauer, & Schlösser, 2013), and (d) *self-esteem* as the well-being indicator (the most appropriate indicator according to most theories on person-culture match; Gebauer et al., 2017). To test for generalizability, we conducted four additional studies (reported in the Supplemental Material available online) that relied on the same data set but used *informant reports* as the reporting method (Study S1), *federal states* within the United States as the units of culture (Study S2), *political liberalism* as the person-culture match domain (Study S3), and *depression* as the well-being indicator (Study S4). By and large, the supplemental studies replicated the results of the main study, attesting to their generalizability and robustness.^1^

## Method

The data came from the Gosling-Potter Internet Personality Project (GPIPP; Gosling, Vazire, Srivastava, & John, 2004). Currently available GPIPP data were collected from December 1998 to March 2015. All published GPIPP research is listed at http://www.thebigfiveproject.com/publishedpapers/.

### Participants

The GPIPP data set comprises data from multiple online studies (the data set underwent several steps of a priori data cleaning; see Section S1 in the Supplemental Material). To extract the relevant data, we applied two standard selection criteria in GPIPP research (Gebauer et al., 2017; Gebauer et al., 2015). First, we selected participants who completed at least one item of each relevant measure (religiosity, self-esteem, Big Two, Big Five). Second, we selected countries with at least 300 participants. The resultant sample contained data from 2,672,820 participants across 102 countries (62.18% women; age: *M* = 25.54 years, *SD* = 10.91). Section S2 in the Supplemental Material includes demographics for each country.

### Procedure

Participants first chose the language of the study (66.50% chose English, 19.33% Spanish, 8.21% German, and 5.96% Dutch). Next, participants completed the following measures (in this order): basic personality traits, self-esteem, religiosity, and demographics. Finally, participants obtained personalized personality feedback and information on personality psychology.

### Measures

All measures used rating scales ranging from 1 (*strongly disagree*) to 5 (*strongly agree*; for means and standard deviations, see Section S3 in the Supplemental Material).

#### Big Five

The 44-item Big Five Inventory (BFI) is the most widely used nonproprietary measure of the Big Five (John, Donahue, & Kentle, 1991). One example item per Big Five trait follows (each item starts with the phrase “I see myself as someone who . . .”): “. . . has a forgiving nature” (agreeableness); “. . . is a reliable worker” (conscientiousness); “. . . has an active imagination” (openness to experience); “. . . is outgoing, sociable” (extra-version); and “. . . worries a lot” (neuroticism). Section S4 in the Supplemental Material reports the number of items per Big Five trait, internal consistencies, and measurement invariances across countries.

#### Big Two

Entringer, Gebauer, and Paulhus (2020) found that communion and agency can be approximated with items from the BFI. To construct the BFI-Big Two Scales, they used four different scale-construction methods: expert rating, target scale, ant colony, and brute force (for a description of those methods, see Entringer et al., 2020). All four methods yielded valid scales (Section S5 in the Supplemental Material describes which BFI items belong to which BFI-Big Two Scale). Among other things, the associations between any of the four BFI-Communion Scales and extant communion scales were comparable with the associations between those extant communion scales and each other. Likewise, the associations between any of the four BFI-Agency Scales and extant agency scales were comparable with the associations between those extant agency scales and each other. Section S4 reports the number of items per Big Two trait, internal consistencies, and measurement invariance across countries for all four BFI-Big Two Scales. Notably, this supplement shows that the scale based on the expert-rating method yielded insufficient measurement invariance. For that reason, we exclusively report the results for the other three BFI-Big Two Scales.

#### Self-esteem

The GPIPP’s variant of the Single-Item Self-Esteem Scale (SISE; Robins, Hendin, & Trzesniewski, 2001) is “I see myself as someone who has high self-esteem.” The SISE’s retest reliability is high (*r* = .75), and the SISE possesses near-perfect dissattenuated correlations with the Rosenberg Self-Esteem Scale (.89 ≤ *r* ≤ .94; Robins et al., 2001).

#### Religiosity

The GPIPP Single-Item Religiosity Scale (SIRS; Entringer et al., in press) is “I see myself as someone who is very religious.” The SIRS’s retest reliability is high (*r* = .92), and the SIRS possesses near-perfect dissattenuated correlations with extant multi-item measures of global religiosity (.96 ≤ *r* ≤ .98; Entringer et al., in press).

#### Country-level religiosity

We averaged participants’ SIRS scores within each country—the standard approach to assess country-level religiosity (Diener et al., 2011; Entringer et al., in press; Gebauer et al., 2017). That average correlated strongly with an external index of country-level religiosity based on Gallup World Poll data, *r*(94) = .86, 95% confidence interval (CI) = [.80, .91] (Joshanloo & Gebauer, 2020). Section S2 includes the present index.

### Statistical modeling

We conducted linear mixed-effects models to account for the nested data structure (participants nested in countries). We used Bates’s (2018) *MixedModels* package within the statistical software environment Julia (Bezanson, Edelman, Karpinski, & Shah, 2017).

To examine person-culture match’s well-being benefits, Fulmer et al. (2010) tested for a cross-level interaction between the Level 1 person variable (here, religiosity) and the Level 2 culture variable (here, country-level religiosity) on well-being (here, self-esteem). This strategy has become the standard for examining religiosity-match effects (Diener et al., 2011; Ebert, Gebauer, Talman, & Rentfrow, 2020; Gebauer, Sedikides, & Neberich, 2012; Gebauer et al., 2017; Stavrova, 2015; Stavrova, Fetchenhauer, & Schlösser, 2013). Consequently, we built on that strategy and tested whether basic personality traits moderated the cross-level interaction between religiosity and country-level religiosity on self-esteem.

In the case of the Big Two, for example, we tested for the simultaneous presence of 2 three-way interactions: (a) Religiosity × Country-Level Religiosity × Communion and (b) Religiosity × Country-Level Religiosity × Agency. Therefore, our Big Two model included self-esteem as the criterion and those 2 three-way interactions as focal predictors. Additional predictors were all main effects and two-way interactions of the variables contained in the 2 three-way interactions (Aiken & West, 1991). We conducted an analogous model for the Big Five (i.e., five simultaneous three-way interactions).

In all our models, we group-mean-centered all Level 1 predictors and grand-mean-centered all Level 2 predictors, which allowed unambiguous interpretation of our cross-level interactions (Enders & Tofighi, 2007). We further *z*-standardized all variables in our models to receive standardized point estimates (*z*PEs), akin to betas in multiple regression (Snijders & Bosker, 2012). Finally, we specified as random all intercepts and Level 1 slopes in our models (Barr, Levy, Scheepers, & Tily, 2013).

We adopted Gebauer et al.’s (2017) three-step approach to estimate the size of the person-culture match effect. First, we estimated the simple slope between religiosity and self-esteem in most religious countries (i.e., we reran the above-described mixed-effects models after recentering country-level religiosity so that the most religious country was set to zero; simple-slopes test; Aiken & West, 1991). Second, we estimated the simple slope between religiosity and self-esteem in least religious countries (i.e., we reran the above-described mixed-effects models after recentering country-level religiosity so that the least religious country was set to zero; simple-slopes test; Aiken & West, 1991). Third, we calculated the difference between those two estimates or simple slopes (∆*z*PE). We calculated the upper confidence limit of ∆*z*PE by taking the difference between the upper confidence limit of the most religious countries’ estimate or simple slope and the lower confidence limit of the least religious countries’ estimate or simple slope. Analogously, we calculated the lower confidence limit of ∆*z*PE by taking the difference between the lower and upper confidence limit, respectively, of the most religious countries’ and the least religious countries’ estimate or simple slope.

Our primary goal was to test whether basic personality traits moderate the person-culture match effect. Hence, we were most interested in the three-way interactions described above. An additional goal was to test whether there are any people who do not benefit at all from person-culture match or who even benefit from mismatch rather than match. To examine that more-exploratory possibility, we adapted Aiken and West’s (1991) recentering approach. Specifically, we reran our above-described mixed-effects models after recentering the personality traits as follows: We recentered them such that the Religiosity × Country-Level Religiosity interaction indicated the size of the person-culture match effect for people with a personality configuration least likely to benefit from person-culture match. In the case of the Big Two, we recentered communion and agency so that the Religiosity × Country-Level Religiosity interaction indicated the size of the person-culture match effect for people who possessed low levels of communion coupled with high levels of agency—that is, for “strong contrasters” within the Big Two framework (Aiken & West, 1991). We tested whether those strong contrasters would still benefit from person-culture match (probably because of the social support they receive; Diener et al., 2011), whether they would not benefit from person-culture match at all, or whether they would even benefit from mismatch rather than match.

## Results

Tables 1 and 2 show the results of our four models (three Big Two models—one per Big Two measure—and the Big Five model). All four models revealed the typical person-culture match effect; namely, Religiosity × Country-Level Religiosity predicted higher self-esteem (Diener et al., 2011; Gebauer, Sedikides, & Neberich, 2012). Decomposition of those two-way interactions via simple-slope tests revealed their precise nature. Across all four models, the positive association between religiosity and self-esteem was very small in the least religious countries, $\bar{zPE}$ = .04, $\bar{95\%\;\mathrm{CI}}$ = [.02, .05]. In the most religious countries, however, the same association was larger, $\bar{zPE}$ = .14, $\bar{95\%\;\mathrm{CI}}$ = [.13, .16].^2^ Thus, the size of the person-culture match effect was Δ$\bar{zPE}$ = .10, $\bar{95\%\;\mathrm{CI}}$ = [.08, .14].

**Table 1.** The Person-Culture Match Effect Moderated by the Big Two

Predictor	Big Two model					
	Target scale		Ant colony		Brute force	
	*z*PE	95% CI	*z*PE	95% CI	*z*PE	95% CI
Intercept	.078	[.056, .101]	.080	[.057, .102]	.079	[.057, .102]
Religiosity	.073	[.066, .080]	.092	[.086, .098]	.083	[.077, .089]
Country-level religiosity	.126	[.104, .149]	.127	[.104, .149]	.127	[.104, .149]
Communion	.070	[.060, .081]	.054	[.045, .063]	.043	[.033, .053]
Agency	.430	[.419, .441]	.448	[.437, .459]	.390	[.381, .399]
Religiosity × Country-Level Religiosity	.025	[.018, .032]	.026	[.020, .032]	.031	[.025, .037]
Religiosity × Communion	.010	[.009, .011]	.004	[.003, .005]	.006	[.005, .007]
Country-Level Religiosity × Communion	.046	[.036, .056]	.028	[.019, .037]	.034	[.024, .044]
Religiosity × Agency	−.010	[−.011, −.009]	−.019	[−.020, −.018]	−.016	[−.017, −.014]
Country-Level Religiosity × Agency	−.029	[−.041, −.018]	−.028	[−.039, −.017]	−.024	[−.033, −.014]
Religiosity × Country-Level Religiosity × Communion	.002	[.001, .004]	.003	[.002, .005]	.004	[.003, .006]
Religiosity × Country-Level Religiosity × Agency	−.007	[−.009, −.006]	−.010	[−.011, −.009]	−.008	[−.010, −.007]

Note: In this model, self-esteem is predicted by religiosity, country-level religiosity, Big Two personality, and their interactions. Results are shown for three variants of the model, one variant for each Big Two measure (target scale, ant colony, brute force). The table shows standardized point estimates (*z*PEs) and 95% confidence intervals (CIs).

**Table 2.** The Person-Culture Match Effect Moderated by the Big Five

Predictor	*z*PE	95% CI
Intercept	.081	[.058, .103]
Religiosity	.083	[.077, .089]
Country-Level Religiosity	.128	[.105, .150]
Agreeableness	−.094	[−.101, −.086]
Conscientiousness	.137	[.131, .143]
Openness	.117	[.109, .125]
Extraversion	.284	[.273, .295]
Neuroticism	−.306	[−.320, −.292]
Religiosity × Country-Level Religiosity	.019	[.013, .025]
Religiosity × Agreeableness	.004	[.003, .005]
Country-Level Religiosity × Agreeableness	.038	[.031, .046]
Religiosity × Conscientiousness	−.004	[−.005, −.003]
Country-Level Religiosity × Conscientiousness	.030	[.023, .036]
Religiosity × Openness	−.007	[−.008, −.006]
Country-Level Religiosity × Openness	.004	[−.004, .012]
Religiosity × Extraversion	−.006	[−.007, −.005]
Country-Level Religiosity × Extraversion	−.024	[−.035, −.013]
Religiosity × Neuroticism	.015	[.013, .016]
Country-Level Religiosity × Neuroticism	.033	[.019, .047]
Religiosity × Country-Level Religiosity × Agreeableness	.003	[.002, .005]
Religiosity × Country-Level Religiosity × Conscientiousness	−.003	[−.004, −.002]
Religiosity × Country-Level Religiosity × Openness	−.004	[−.006, −.003]
Religiosity × Country-Level Religiosity × Extraversion	−.005	[−.006, −.003]
Religiosity × Country-Level Religiosity × Neuroticism	.007	[.005, .008]

Note: In this model, self-esteem is predicted by religiosity, country-level religiosity, Big Five personality, and their interactions. The table shows standardized point estimates (*z*PEs) and 95% confidence intervals (CIs).

Most relevant for our research question, results for all four models also revealed that basic personality traits moderated the person-culture match effect. In the Big Two models (Table 1), communion exacerbated the person-culture match effect (i.e., Religiosity × Country-Level Religiosity × Communion predicted higher self-esteem) and agency diminished it (i.e., Religiosity × Country-Level Religiosity × Agency predicted lower self-esteem).

**Fig. 1.**
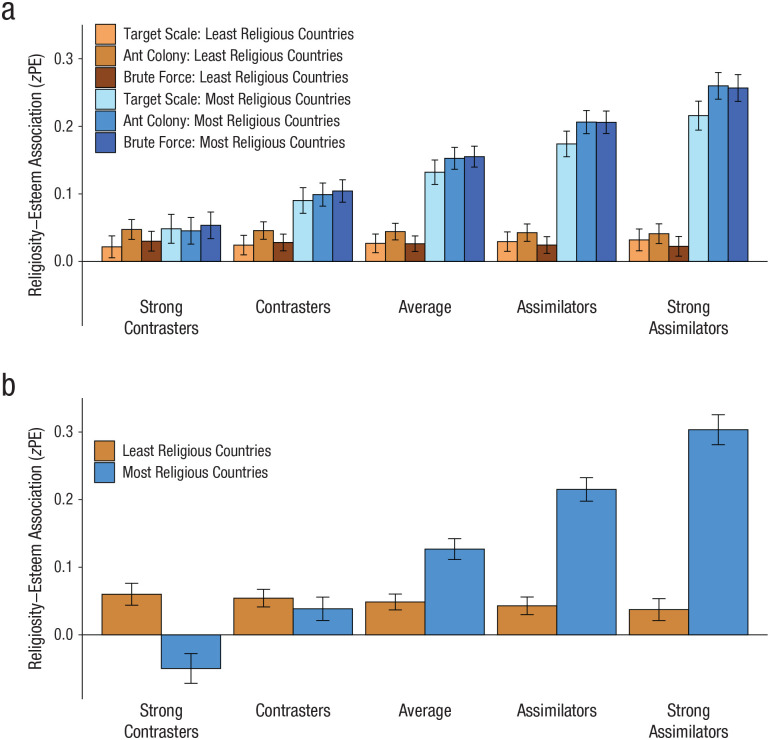
Person-culture match effect: mean standardized point estimate (*z*PE) for the association between religiosity and self-esteem, separately for strong contrasters, contrasters, the average participant, assimilators, and strong assimilators. Results are shown in (a) for each group within the Big Two framework, separately for each of the three scale-construction methods and for groups in least and most religious countries. Results are shown in (b) for each group within the Big Five framework, separately for groups in least and most religious countries. Contrasters were defined as individuals +1 *SD* on all contrast-eliciting traits and –1 *SD* on all assimilation-eliciting traits, and strong contrasters were defined as individuals +2 *SD* and –2 *SD* on those respective traits. Assimilators were defined as individuals +1 *SD* on all assimilation-eliciting traits and –1 *SD* on all contrast-eliciting traits, and strong assimilators were defined as individuals +2 *SD* and –2 *SD* on those respective traits. Error bars indicate 95% confidence intervals.

In addition, Figure 1a shows that the size of the person-culture match effect was considerably diminished among strong contrasters (low communion and high agency) across all three scale-construction methods—target scale: ∆*z*PE = .03, 95% CI = [−.01, .06]; ant colony: ∆*z*PE = −.002, 95% CI = [−.04, .03]; brute force: ∆*z*PE = .02, 95% CI = [−.01, .06]. In fact, strong contrasters showed no significant person-culture match effect. By contrast, the size of the person-culture match effect was considerably exacerbated among strong assimilators (high communion and low agency)—target scale: ∆*z*PE = .18, 95% CI = [.15, .22]; ant colony: ∆*z*PE = .22, 95% CI = [.18, .25]; brute force: ∆*z*PE = .23, 95% CI = [.20, .27]. Evidently, the person-culture match effect can be much more powerful than previously thought (Diener et al., 2011; Gebauer et al., 2017; Stavrova, Fetchenhauer, & Schlösser, 2013).

In the Big Five model (Table 2), agreeableness and neuroticism exacerbated the person-culture match effect, whereas openness to experience, extraversion, and conscientiousness diminished it. We did not predict the exacerbating effect of neuroticism. In hindsight, however, that effect is consistent with previous evidence that neuroticism is positively associated with concern for appropriate behavior, including norm-conforming behavior (Lennox & Wolfe, 1984). We also did not predict the diminishing effect of extraversion. Yet it is consistent with extraverts’ agentic nature (Paulhus & John, 1998). Finally, the diminishing effect of conscientiousness ran counter to our predictions, but this effect is consistent with a close connection between conscientiousness and “getting ahead” (i.e., agency goals; Roberts & Robins, 2000).

In addition, Figure 1b shows that the size of the person-culture match effect was considerably diminished among strong contrasters (low agreeableness and neuroticism coupled with high openness, extraversion, and conscientiousness; ∆*z*PE = −.11, 95% CI = [−.15, −.07]). In fact, strong contrasters within the Big Five framework showed a reversed person-culture match effect. This pattern suggests that strong contrasters may actually suffer well-being costs as a result of person-culture match. By contrast, the size of the person-culture match effect was considerably exacerbated among strong assimilators (high agreeableness and neuroticism coupled with low openness, extraversion, and conscientiousness; ∆*z*PE = .27, 95% CI = [.23, .30]). This, again, illustrates that the person-culture match effect can be much more powerful than previously thought.

In all, our four models clearly showed that basic personality traits moderate the benefits of person-culture match on well-being. But how powerful was that moderation? Perhaps the most telling way to answer this question is to compare the power of personality with the indisputable power of culture. The power of culture can be estimated as the extent to which culture alters the association between religiosity and self-esteem. This amount is given by the size of the person-culture match effect. Analogously, the power of personality can be estimated as the extent to which personality alters the person-culture match effect. That amount is given by the difference in the person-culture match effect between strong assimilators and strong contrasters. In the case of the Big Two, the power of culture was .11 (when the Big Two scales were constructed with the target-scale method), .11 (when the Big Two scales were constructed with the ant-colony method), and .13 (when the Big Two scales were constructed with the brute-force method), and the power of personality was .15 (target scale), .22 (ant colony), and .21 (brute force). In the case of the Big Five, the power of culture was .08, and the power of personality was .38. Evidently, the power of personality surpassed the power of culture.

Studies S1 to S4 probed the generalizability of our main results. Study S1 shows that the main results were replicated nearly perfectly even though we avoided using self-reports altogether and instead relied on informant reports of all variables in our mixed-effects models. Evidently, biases in self-reports cannot explain our results. Study S2 shows that the main results were largely replicated even when federal states served as cultural units. Evidently, our findings extend to cultural units other than countries (but see Study S2 for some restrictions to this extension). Study S3 shows that the main results also were largely replicated even when political liberalism served as the domain for person-culture match (but see the Big Five model as an exception). Evidently, our findings extend to match domains other than religiosity. Finally, Study S4 shows that the main results were largely replicated even when depression served as the well-being indicator. Evidently, our findings extend to well-being indicators other than self-esteem. By and large, then, the results of Studies S1 to S4 buttress the main results. As a whole, these results provide strong evidence for our key claim that the Big Two and the Big Five moderate person-culture match’s well-being benefits.

## Discussion

The person-culture match effect is a classic in psychology (Oishi, Diener, Suh, & Lucas, 1999) and—as a driver of migration—has huge societal implications (Jokela et al., 2008). But do individuals differ in the degree to which person-culture match benefits their well-being? And, if so, might some people benefit not at all from person-culture match? Or might some even benefit from mismatch? We extended prior theory and research on the Big Two (Gebauer, Leary, & Neberich, 2012; Gebauer, Paulhus, & Neberich, 2013; Gebauer, Sedikides, et al., 2014) and the Big Five (Ashton & Lee, 2019; Entringer et al., in press; Gebauer, Bleidorn, et al., 2014) and predicted that both basic personality taxonomies should include moderators of the person-culture match effect.

The main study tested our predictions in a sample of 2,672,820 people across 102 countries. For good reason (see the introduction), that study used self-reports as the reporting method, countries as the cultural units, religiosity as the match domain, and self-esteem as the well-being indicator. Four supplemental studies used informant reports as the reporting method (Study S1), U.S. states as the cultural units (Study S2), political liberalism as the match domain (Study S3), and depression as the well-being indicator (Study S4).

Considering their differences, we found that the five studies revealed very consistent results. Within the Big Two framework, communion exacerbated the person-culture match effect and agency diminished it (the few exceptions are noted in Studies S1–S4, in which a relevant three-way interaction did not reach significance). Within the Big Five framework, agreeableness and neuroticism exacerbated the person-culture match effect, and openness, extraversion, and conscientiousness diminished it (again, exceptions are noted in Studies S1–S4). We did not predict the results regarding neuroticism, extraversion, and conscientiousness, but we consider them highly informative because they fitted other established theories (Lennox & Wolfe, 1984; Paulhus & John, 1998; Roberts & Robins, 2000), rest on large-scale data (2,672,820 people across 102 countries), and were widely replicated (see the main study and four supplemental studies).

How powerful was personality as a moderator of the person-culture match effect? Two tests suggested that personality was powerful. First, the person-culture match effect usually vanished altogether among strong contrasters (Fig. 1 and Figs. S1–S4), and this was the case irrespective of the personality taxonomy used to define strong contrasters (Big Two, Big Five). In fact, we occasionally found that strong contrasters even appeared to suffer from high person-culture match. Second, we compared the power of personality with the power of culture and found that the former generally surpassed the latter.

We note four limitations of our studies that suggest avenues for future research. First, religiosity is the classic match domain, in which person-culture match has been documented most thoroughly (Stavrova, Fetchenhauer, & Schlösser, 2013). However, religiosity is an asymmetrical match domain: In religious cultures, religious people experience a feeling of match. But in nonreligious cultures, nonreligious people do not experience such a feeling, because the absence of religiosity is not a salient issue in nonreligious cultures (Gebauer et al., 2017). Likewise, in Study S3, political liberalism was also an asymmetrical match domain, because our particular measure assessed high versus low liberalism rather than liberalism versus conservatism. Future research should replicate our results with symmetrical match domains and, accordingly, should use adapted statistical models (i.e., response-surface analyses; see Bleidorn et al., 2016).

Second, the GPIPP is an opportunity sample of online volunteers and, thus, not nationally representative (Gosling et al., 2004). Yet we have no theoretical reason to suspect that this nonrepresentativeness spuriously caused our results. Further ameliorating representativeness concerns, the person-culture match effect in the domain of religiosity replicates across the GPIPP (Gebauer et al., 2017) and the world-representative Gallup World Poll (Diener et al., 2011). Moreover, the robustness and generalizability of our main results are reassuring because they suggest that the results do not hinge on specifics of our main sample (i.e., self-reports as reporting method, countries as cultural units, religiosity as match domain, and self-esteem as well-being indicator). Nonetheless, replication attempts with nation-representative data would be particularly valuable.

Third, we expected basic personality traits (Big Two and Big Five) to moderate the person-culture match effect because those basic traits elicit motives to assimilate to and contrast from ambient norms (Big Two: Gebauer, Leary, & Neberich, 2012; Gebauer et al., 2013; Gebauer, Sedikides, et al., 2014; Big Five: Ashton & Lee, 2019; Entringer et al., in press; Gebauer, Bleidorn, et al., 2014). Yet empirical research is scarce on why basic personality traits elicit assimilation and contrast motives. This issue needs to be attended to in future research.

Finally, match effects on well-being not only occur between people and their cultures (person-culture match) but also between people and their spouses, parents, friends, colleagues, coworkers, organizations, and so on (Kristof-Brown & Guay, 2011). Future research should test whether basic personality traits also moderate those match effects. Our findings already generalized across countries and across U.S. states (Study S2). Thus, we see little reason that our findings should not extend to even more fine-grained units of match as well.

The present research has major implications. First, many psychological theories build on the person-culture match effect (for a review, see Gebauer et al., 2015). According to our results, those theories may profit from incorporating personality differences. Second, few explanations exist for why person-culture match confers well-being benefits (Diener et al., 2011; Fulmer et al., 2010; Gebauer et al., 2017). The present research adds a novel explanation. Specifically, personality traits that exacerbate the person-culture match effect are more pronounced than personality traits that diminish the effect (e.g., people typically are more communal than agentic; Allison, Messick, & Goethals, 1989). This is probably why there is an overall person-culture match effect (accompanied by pronounced personality differences in its size). Finally, evidence for the person-culture match effect has been inconsistent in the literature (Bleidorn et al., 2016). The present research suggests an explanation for that inconsistency. According to our research, the evidence should vary between samples as a function of their personality composition.

In conclusion, the present research (main study and four supplemental studies) provides ample evidence that the person-culture match effect is contingent on basic personality traits (Big Two and Big Five). That research is conceptually innovative, empirically novel, and highly consequential for basic psychology and for societal challenges alike.

## Supplemental Material

Gebauer_OpenPracticesDisclosure_rev – Supplemental material for The Well-Being Benefits of Person-Culture Match Are Contingent on Basic Personality Traits Click here for additional data file. Supplemental material, Gebauer_OpenPracticesDisclosure_rev for The Well-Being Benefits of Person-Culture Match Are Contingent on Basic Personality Traits by Jochen E. Gebauer, Jennifer Eck, Theresa M. Entringer, Wiebke Bleidorn, Peter J. Rentfrow, Jeff Potter and Samuel D. Gosling in Psychological Science

Gebauer_Supplemental_Material_rev – Supplemental material for The Well-Being Benefits of Person-Culture Match Are Contingent on Basic Personality Traits Click here for additional data file. Supplemental material, Gebauer_Supplemental_Material_rev for The Well-Being Benefits of Person-Culture Match Are Contingent on Basic Personality Traits by Jochen E. Gebauer, Jennifer Eck, Theresa M. Entringer, Wiebke Bleidorn, Peter J. Rentfrow, Jeff Potter and Samuel D. Gosling in Psychological Science

## Transparency

*Action Editor:* Ayse K. Uskul

*Editor:* D. Stephen Lindsay

*Author Contributions*

J. E. Gebauer developed the study concept. J. Eck and J. E. Gebauer analyzed and interpreted the data. J. E. Gebauer drafted the manuscript with J. Eck’s help. All of the authors provided critical revisions and approved the final manuscript for submission.

**Open Practices:** The University of Mannheim Research Data Repository (MADATA) provides deidentified data and data-analysis scripts (https://madata.bib.uni-mannheim.de/330/) as well as study materials (http://madata.bib.uni-mannheim.de/331/), all of which are publicly available. The design and analysis plans for the present study were not preregistered. The complete Open Practices Disclosure for this article can be found at http://journals.sagepub.com/doi/suppl/10.1177/0956797620951115. This article has received the badges for Open Data and Open Materials. More information about the Open Practices badges can be found at http://www.psychologicalscience.org/publications/badges.

## Notes

1. We did not preregister our analyses, but we strictly followed published recommendations on how to statistically test our hypotheses (Fulmer et al., 2010; Gebauer et al., 2017; Stavrova, Fetchenhauer, & Schlösser, 2013; Stavrova, Schlösser, & Fetchenhauer, 2013).

2. This finding is partly a replication and partly a reproduction of Gebauer et al.’s (2017) Study 1, which also relied on the GPIPP data set, albeit on an earlier version with fewer participants (2,195,301 vs. 2,672,820) and countries (65 vs. 102).
