# Supplementary material for: The Well-Being Benefits of Person-Culture Match Are Contingent on Basic Personality Traits
Source: Psychol Sci. 2020 Sep 14;31(10):1283–93. doi: 10.1177/0956797620951115 (PMC7549288; doi:10.1177/0956797620951115)
Supplement: Gebauer_Supplemental_Material_rev – Supplemental material for The Well-Being Benefits of Person-Culture Match Are Contingent on Basic Personality Traits [file Gebauer_Supplemental_Material_rev.docx]

**Supplemental Material**

**Study S1.** The main text focuses on self-reports for good reason: self-reports are the most commonly used reporting method in social-personality psychology (Paulhus & Vazire, 2007) and they can be more valid than informant-reports (Vazire, 2010). Of course, informant-reports have their strengths, too (Vazire, 2010). Above all, informant-reports circumvent self-report biases (Paulhus & Vazire, 2007). Our main-text results would, therefore, be greatly buttressed if they replicated in informant-reports. Consequently, we repeated all main-text analyses, using informant-reports of all the constructs involved (i.e., religiosity, country-level religiosity, self-esteem, Big Two, Big Five).

The GPIPP again provided the data. A subset of GPIPP participants completed the main-text measures but reported on “someone whom you know well, such as a close friend, coworker, or family member” (for similar approaches, see Funder & Colvin, 1988; Hogan et al., 1996; Vazire & Mehl, 2008). To extract the relevant data, we applied the two selection criteria from the main text. The resultant sample contained data from 850,877 informants across 61 countries (64.35% women, *M*_age_ = 26.09 years, *SD*_age_ = 11.07; questionnaire language: 37.75% English, 39.05% Spanish, 11.46% German, 11.75% Dutch). The measures’ psychometric properties were similar to the psychometric properties reported in the main text.^S1^ Our informant-report country-level religiosity measure correlated strongly with an external country-level religiosity index based on Gallup World Poll data, *r*(57) = .89, 95% CI [.81, .93] (Joshanloo & Gebauer, 2020).

We used the exact same statistical modeling strategy as that used in the main text. Table S1 includes the results of the four models (three Big Two models and the Big Five model). The table reveals that basic personality traits again moderated person-culture match’s well-being benefits. Specifically, the informant-report results largely replicated the self-report results from the main text. Notably, though, three of the 11 three-way interactions that were significant in self-report did not reach significance in informant-report. A comparison of Fig. 1 and S1 is perhaps most telling regarding the overall similarity between the informant- and self-report results. The two figures are highly similar. Such high similarity is not trivial at all because informant-reports and self-reports are subject to very different biases (Paulhus & Vazire, 2007; Vazire, 2010).

**Table S1.** Informant-Report Results of the Person-Culture Match Effect Moderated by the Big Two and the Big Five

| **Big Two** | | | | | | | | |  | **Big Five** | | |
| --- | --- | --- | --- | --- | --- | --- | --- | --- | --- | --- | --- | --- |
|  | **TS** | |  | **AC** | |  | **BF** | |  |  |  |  |
|  | ***zPE*** | **95% CI** |  | ***zPE*** | **95% CI** |  | ***zPE*** | **95% CI** |  |  | ***zPE*** | **95% CI** |
| (intercept) | .052 | [.028, .076] |  | .053 | [.029, .077] |  | .052 | [.028, .076] |  | (intercept) | .053 | [.029, .077] |
| rel | .040 | [.034, .046] |  | .065 | [.059, .070] |  | .047 | [.041, .052] |  | rel | .054 | [.048, .059] |
| cnt rel | .089 | [.065, .113] |  | .090 | [.066, .114] |  | .089 | [.065, .113] |  | cnt rel | .090 | [.066, .114] |
| com | .066 | [.050, .081] |  | .015 | [.001, .029] |  | .004 | [-.013, .020] |  | agr | -.111 | [-.122, -.100] |
| agy | .388 | [.374, .401] |  | .411 | [.399, .423] |  | .348 | [.338, .359] |  | cns | .126 | [.119, .133] |
| rel x cnt rel | .021 | [.015, .027] |  | .023 | [.018, .029] |  | .024 | [.018, .030] |  | opn | .114 | [.105, .124] |
| rel x com | .012 | [.010, .014] |  | .004 | [.002, .006] |  | .008 | [.006, .010] |  | ext | .296 | [.281, .312] |
| cnt rel x com | .051 | [.036, .066] |  | .031 | [.017, .044] |  | .048 | [.031, .064] |  | neu | -.271 | [-.285, -.258] |
| rel x agy | -.004 | [-.006, -.002] |  | -.006 | [-.008, -.005] |  | .001 | [-.001, .003] |  | rel x cnt rel | .014 | [.008, .019] |
| cnt rel x agy | -.023 | [-.037, -.010] |  | -.026 | [-.038, -.014] |  | -.014 | [-.024, -.004] |  | rel x agr | .008 | [.006, .010] |
| rel x cnt rel x com | .002 | [-.001, .004] |  | .001 | [-.001, .004] |  | .003 | [.001, .006] |  | cnt rel x agr | .044 | [.033, .055] |
| rel x cnt rel x agy | -.008 | [-.010, -.005] |  | -.006 | [-.009, -.004] |  | -.006 | [-.009, -.004] |  | rel x cns | -.003 | [-.005, -.001] |
|  |  |  |  |  |  |  |  |  |  | cnt rel x cns | .030 | [.023, .037] |
|  |  |  |  |  |  |  |  |  |  | rel x opn | .010 | [.008, .012] |
|  |  |  |  |  |  |  |  |  |  | cnt rel x opn | .035 | [.025, .044] |
|  |  |  |  |  |  |  |  |  |  | rel x ext | -.008 | [-.010, -.006] |
|  |  |  |  |  |  |  |  |  |  | cnt rel x ext | -.023 | [-.038, -.008] |
|  |  |  |  |  |  |  |  |  |  | rel x neu | .014 | [.012, .016] |
|  |  |  |  |  |  |  |  |  |  | cnt rel x neu | .048 | [.035, .061] |
|  |  |  |  |  |  |  |  |  |  | rel x cnt rel x agr | .003 | [.001, .005] |
|  |  |  |  |  |  |  |  |  |  | rel x cnt rel x cns | -.005 | [-.007, -.002] |
|  |  |  |  |  |  |  |  |  |  | rel x cnt rel x opn | -.001 | [-.004, .001] |
|  |  |  |  |  |  |  |  |  |  | rel x cnt rel x ext | -.006 | [-.008, -.004] |
|  |  |  |  |  |  |  |  |  |  | rel x cnt rel x neu | .005 | [.003, .008] |

Note: TS = target scale, AC = ant colony, BF = brute force, rel = religiosity, cnt rel = country-level religiosity, agy = agency, com = communion, agr = agreeableness, cns = conscientiousness, ext = extraversion, opn = openness, neu = neuroticism.

**
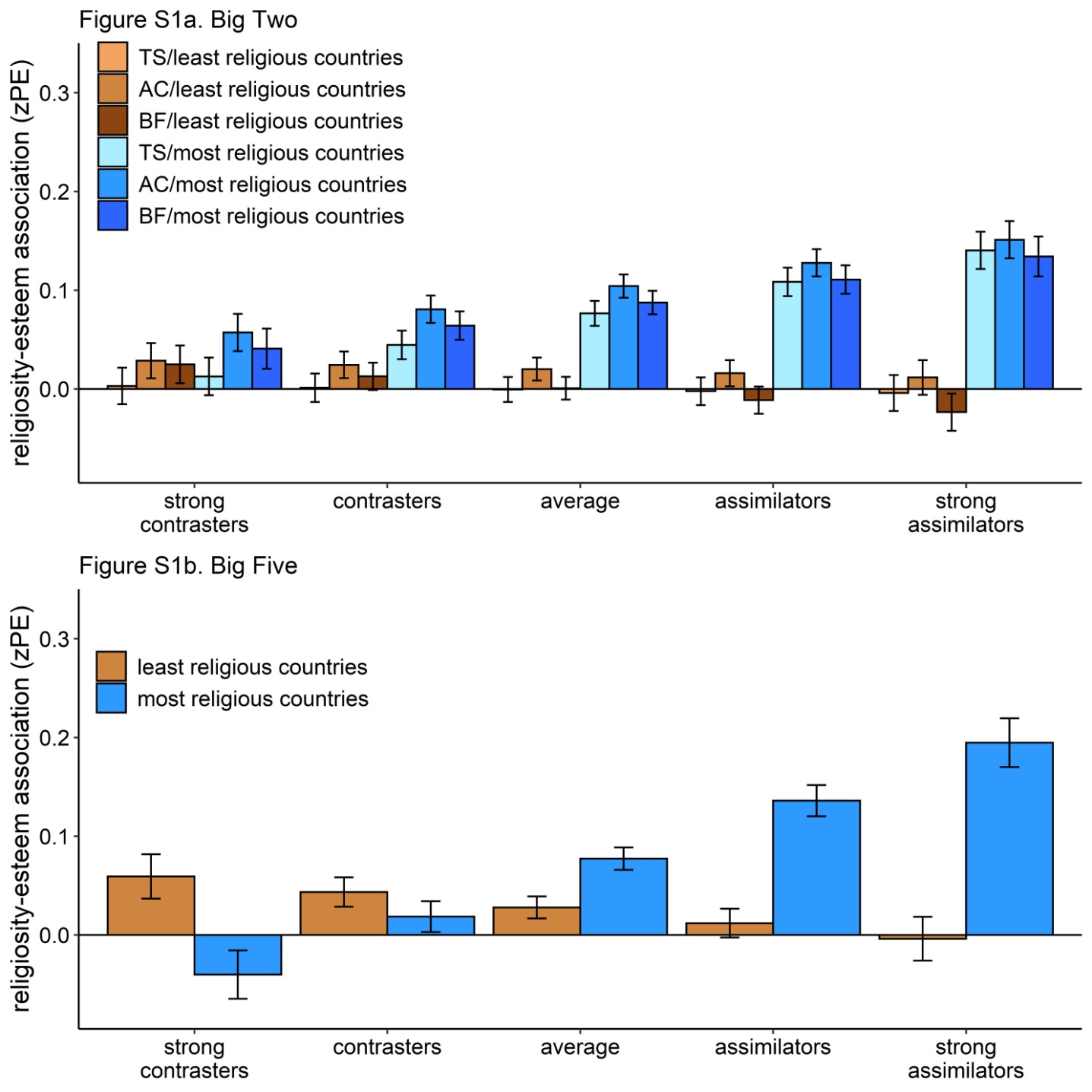
**

**Fig. S1.** Person-culture match effect for assimilators and contrasters in informant-reports. (Strong) assimilators = mean + 1 SD (+ 2 SD) on all assimilation-eliciting traits and mean - 1 SD (-2 SD) on all contrast-eliciting traits, (strong) contrasters = mean + 1 SD (+ 2 SD) on all contrast-eliciting traits and mean - 1 SD (-2 SD) on all assimilation-eliciting traits, TS = target scale, AC = ant colony, BF = brute force. Error bars indicate 95% confidence intervals.

**Study S2.** In the main text, countries are our cultural units. Countries are the most appropriate cultural units because they typically are psychologically most salient and they certainly are the most widely used units of culture (Inglehart & Baker, 2000). However, countries are not the only psychologically relevant units of culture (Talhelm et al., 2014). In addition to countries, federal states are also relevant (Rentfrow et al., 2008). We, thus, examined whether the main-text results generalize to the (federal) state level. Specifically, we once again drew on GPIPP data and tested whether the main-text results replicated within the U.S. with the U.S. states as units of culture. We used the U.S. for two reasons: (1) largest sample size within the GPIPP, (2) large number of federal states per country (50 states + District of Columbia).

To extract the relevant data, we adapted the main text’s two selection criteria: (1) participants with at least one completed item of each relevant measure (i.e., religiosity, self-esteem, Big Two, Big Five), (2) U.S. states with at least 300 participants (all U.S. states met this criterion). The resultant sample contained data from 1,142,224 participants across 51 U.S. states (61.43% women, *M*_age_ = 25.11 years, *SD*_age_ = 11.12; questionnaire language: 99.47% English, 0.48% Spanish, 0.05% German). The measures’ psychometric properties were at least as satisfactory as the psychometric properties reported in the main text.^S2^ Our state-level religiosity measure correlated strongly with an external state-level religiosity index based on data from the 2009 Gallup U.S. Poll, *r*(49) = .91, 95% CI [.85, .95] (Diener, Tay, & Myers, 2011).

We used the exact same statistical modeling strategy as that used in the main text. Table S2 includes the results of the four models (three Big Two models, Big Five model). The table reveals that basic personality traits once more moderated person-culture match’s well-being benefits. Specifically, most of the main-text results replicated, but there was an exception. Regarding the Big Five, only agreeableness and extraversion significantly moderated the person-culture match effect. However, the main-text results did replicate fully and firmly regarding the Big Two. Importantly, Fig. S2 shows that the overall result pattern was very similar to the main text’s pattern (Fig. 1) and this was the case for both personality taxonomies. It is also important to note that the present results and the main-text results provide information about very different kinds of cultural units (federal states vs. countries). What the present results show is that the Big Two (but less so the Big Five) possess the power to alter the well-being benefits of person-*state* match *independent of* (i.e., *over and above*) their power to alter the well-being benefits of person-*country* match (see main text and Study S1). Put differently, in concert with each other, the two sets of results show that the moderating power of basic personality traits is even larger than suggested by the main-text results alone.

**Table S2.** The Person-Culture Match Effect in U.S. Federal States Moderated by the Big Two and the Big Five

| **Big Two** | | | | | | | | |  | **Big Five** | | |
| --- | --- | --- | --- | --- | --- | --- | --- | --- | --- | --- | --- | --- |
|  | **TS** | |  | **AC** | |  | **BF** | |  |  |  |  |
|  | ***zPE*** | **95% CI** |  | ***zPE*** | **95% CI** |  | ***zPE*** | **95% CI** |  |  | ***zPE*** | **95% CI** |
| (intercept) | -.007 | [-.019, .005] |  | -.006 | [-.018, .006] |  | -.006 | [-.018, .006] |  | (intercept) | -.004 | [-.016, .008] |
| rel | .081 | [.078, .084] |  | .094 | [.091, .097] |  | .095 | [.091, .098] |  | rel | .076 | [.073, .079] |
| sta rel | .030 | [.018, .042] |  | .029 | [.018, .041] |  | .030 | [.018, .042] |  | sta rel | .030 | [.018, .042] |
| com | .121 | [.118, .124] |  | .132 | [.129, .134] |  | .103 | [.100, .106] |  | agr | -.064 | [-.067, -.061] |
| agy | .413 | [.410, .416] |  | .440 | [.436, .443] |  | .369 | [.366, .372] |  | cns | .130 | [.128, .132] |
| rel x sta rel | .010 | [.007, .014] |  | .014 | [.010, .017] |  | .017 | [.013, .021] |  | opn | .061 | [.058, .063] |
| rel x com | .008 | [.006, .010] |  | .003 | [.002, .005] |  | .004 | [.003, .006] |  | ext | .274 | [.270, .277] |
| sta rel x com | .004 | [.001, .006] |  | .005 | [.002, .008] |  | .005 | [.002, .009] |  | neu | -.418 | [-.422, -.414] |
| rel x agy | -.014 | [-.015, -.012] |  | -.024 | [-.026, -.022] |  | -.020 | [-.021, -.018] |  | rel x sta rel | .006 | [.003, .009] |
| sta rel x agy | -.006 | [-.009, -.002] |  | -.006 | [-.009, -.002] |  | -.008 | [-.011, -.004] |  | rel x agr | .003 | [.001, .004] |
| rel x sta rel x com | .003 | [.001, .005] |  | .004 | [.002, .005] |  | .003 | [.001, .005] |  | sta rel x agr | .007 | [.004, .010] |
| rel x sta rel x agy | -.003 | [-.005, -.001] |  | -.003 | [-.005, -.001] |  | -.005 | [-.006, -.003] |  | rel x cns | -.007 | [-.009, -.006] |
|  |  |  |  |  |  |  |  |  |  | sta rel x cns | -.001 | [-.004, .001] |
|  |  |  |  |  |  |  |  |  |  | rel x opn | -.010 | [-.011, -.008] |
|  |  |  |  |  |  |  |  |  |  | sta rel x opn | -.005 | [-.008, -.002] |
|  |  |  |  |  |  |  |  |  |  | rel x ext | -.007 | [-.009, -.006] |
|  |  |  |  |  |  |  |  |  |  | sta rel x ext | -.002 | [-.006, .001] |
|  |  |  |  |  |  |  |  |  |  | rel x neu | .016 | [.014, .017] |
|  |  |  |  |  |  |  |  |  |  | sta rel x neu | .003 | [-.001, .007] |
|  |  |  |  |  |  |  |  |  |  | rel x sta rel x agr | .002 | [2e-05, .004] |
|  |  |  |  |  |  |  |  |  |  | rel x sta rel x cns | -3e-04 | [-.002, .002] |
|  |  |  |  |  |  |  |  |  |  | rel x sta rel x opn | -.001 | [-.003, 4e-04] |
|  |  |  |  |  |  |  |  |  |  | rel x sta rel x ext | -.002 | [-.004, -4e-04] |
|  |  |  |  |  |  |  |  |  |  | rel x sta rel x neu | .001 | [-.001, .003] |

Note: TS = target scale, AC = ant colony, BF = brute force, rel = religiosity, sta rel = state-level religiosity, agy = agency, com = communion, agr = agreeableness, cns = conscientiousness, ext = extraversion, opn = openness, neu = neuroticism.


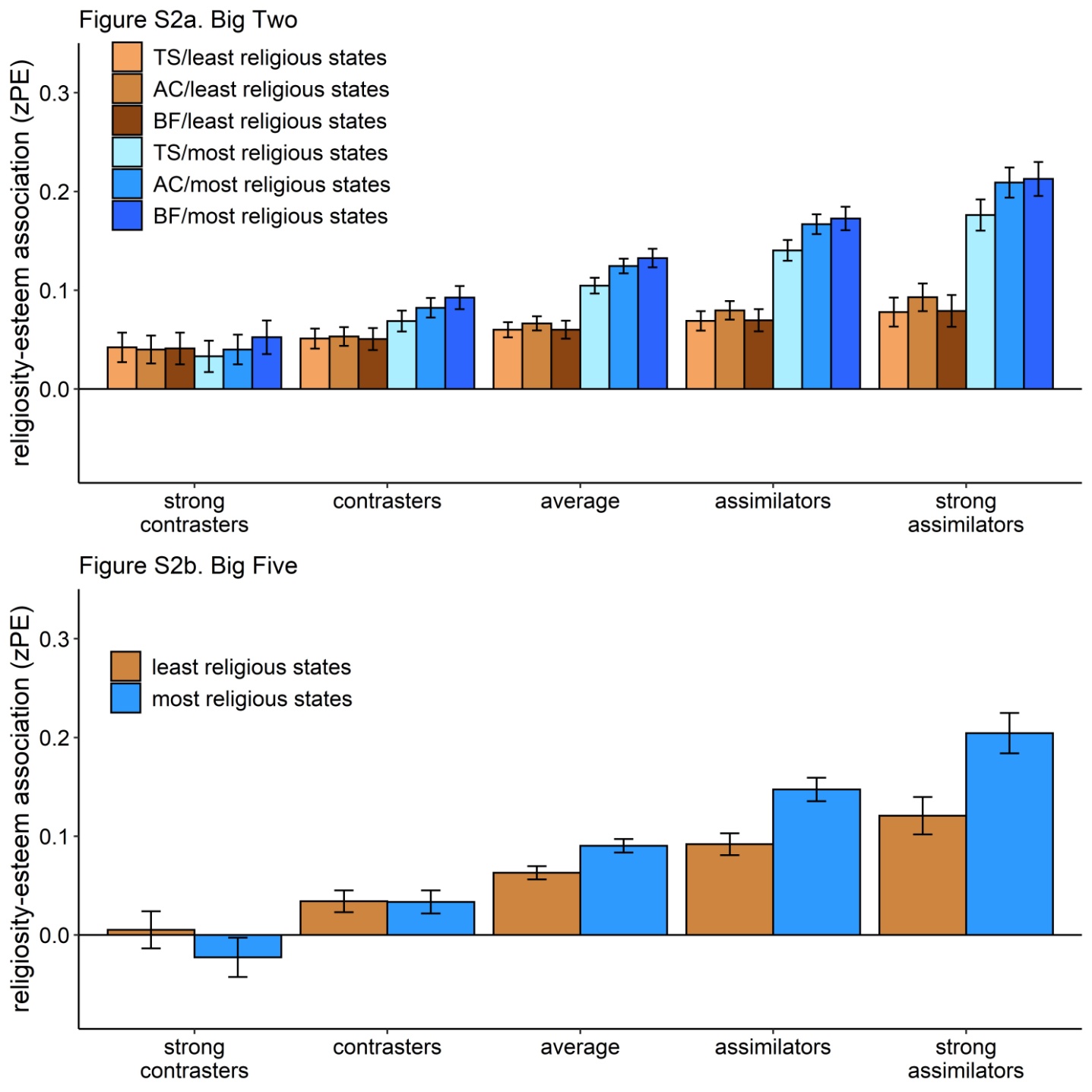


**Fig. S2.** Person-culture match effect for assimilators and contrasters in U.S. federal states. (Strong) assimilators = mean + 1 SD (+ 2 SD) on all assimilation-eliciting traits and mean - 1 SD (-2 SD) on all contrast-eliciting traits, (strong) contrasters = mean + 1 SD (+ 2 SD) on all contrast-eliciting traits and mean - 1 SD (-2 SD) on all assimilation-eliciting traits, TS = target scale, AC = ant colony, BF = brute force. Error bars indicate 95% confidence intervals.

**Study S3.** Religiosity is the match domain used in the main text. Religiosity is ideal because it is the classic match domain (Rosenberg, 1965) and probably the domain in which person-culture match is best documented (Gebauer et al., 2017; Stavrova et al., 2013). Nonetheless, we sought to test the generalizability of our main-text results to other match domains. For that purpose, the GPIPP includes only one viable alternative to religiosity: political liberalism (“I see myself as someone who is politically liberal;” 1 = *strongly disagree*, 5 = *strongly agree*). The term “political liberalism” means different things in different countries (e.g., the term denotes left-wing political views in the U.S., but preliminary right-wing views in Germany). Therefore, it was necessary to restrict our analyses to a single country and to conceptualize person-culture match as the match between people and their *federal state* within that country (as we have already done in Study S2). We had three reasons to choose the U.S. as our focal country: (1) largest sample size within the GPIPP, (2) large number of federal states per country (50 states + District of Columbia), and (3) culturally well-defined meaning of the term “political liberalism.”

To extract the relevant data, we adapted the main text’s two selection criteria: (1) participants with at least one completed item of each relevant measure (i.e., liberalism, self-esteem, Big Two, Big Five), (2) U.S. states with at least 300 participants (all U.S. states met this criterion). The resultant sample contained data from 1,349,487 participants across 51 U.S. states (61.89% women, *M*_age_ = 25.15 years, *SD*_age_ = 11.23; questionnaire language: 99.55% English, 0.41% Spanish, 0.04% German). The measures’ psychometric properties were at least as satisfactory as the psychometric properties reported in the main text.^S3^ Our state-level liberalism measure correlated strongly with an external state-level liberalism index based on Gallup data, *r*(48) = .93, 95% CI [.87, .96] (Gallup, 2018).

We used the exact same statistical modeling strategy as that used in the main text. Table S3 includes the results of the four models (three Big Two models, Big Five model). The table reveals that basic personality traits once again moderated person-culture match’s well-being benefits. Notably, though, the hypotheses were not equally met for both personality taxonomies. They were met partly for the Big Two. For the Big Five, however, they were largely unmet. Having said that, Fig. S3 shows that the overall pattern of the present results is similar to the results in the main text (Fig. 1) and in the other online supplements (Fig. S1, S2, S4) and this was the case for both personality taxonomies. Moreover, we were not surprised that the Big Five results were weaker than the results of the Big Two. After all, Study S2 (cultural units: federal states, but match domain: religiosity) also revealed the weakest results for the Big Five. It, therefore, seems that (except for neuroticism) differences between the present results and the main-text results are better explained by differences in the cultural units (federal states vs. countries) than by differences in the match domain (liberalism vs. religiosity). In other words, the present results (in tandem with the results of Study S2) suggest that our main-text findings are not restricted to religiosity as the sole match domain.

**Table S3.** The Person-Culture Match Effect in the Domain of Political Liberalism Moderated by the Big Two and the Big Five

| **Big Two** | | | | | | | | |  | **Big Five** | | |
| --- | --- | --- | --- | --- | --- | --- | --- | --- | --- | --- | --- | --- |
|  | **TS** | |  | **AC** | |  | **BF** | |  |  |  |  |
|  | ***zPE*** | **95% CI** |  | ***zPE*** | **95% CI** |  | ***zPE*** | **95% CI** |  |  | ***zPE*** | **95% CI** |
| (intercept) | -.006 | [-.021, .008] |  | -.006 | [-.020, .008] |  | -.006 | [-.021, .008] |  | (intercept) | -.005 | [-.020, .009] |
| lib | .016 | [.014, .018] |  | -.003 | [-.006, -.001] |  | -.033 | [-.035, -.030] |  | lib | .025 | [.023, .026] |
| sta lib | -.011 | [-.026, .003] |  | -.011 | [-.026, .003] |  | -.011 | [-.026, .003] |  | sta lib | -.011 | [-.026, .003] |
| com | .135 | [.132, .138] |  | .150 | [.147, .153] |  | .122 | [.118, .125] |  | agr | -.054 | [-.056, -.051] |
| agy | .420 | [.417, .423] |  | .444 | [.441, .447] |  | .379 | [.376, .381] |  | cns | .138 | [.135, .140] |
| lib x sta lib | .001 | [-.002, .004] |  | -.002 | [-.005, .001] |  | -.002 | [-.005, .001] |  | opn | .053 | [.051, .056] |
| lib x com | .008 | [.007, .010] |  | .006 | [.004, .007] |  | .007 | [.006, .009] |  | ext | .277 | [.274, .280] |
| sta lib x com | -.006 | [-.009, -.002] |  | -.009 | [-.012, -.006] |  | -.010 | [-.014, -.006] |  | neu | -.421 | [-.425, -.416] |
| lib x agy | .002 | [.001, .004] |  | .001 | [-.001, .002] |  | .004 | [.002, .005] |  | lib x sta lib | -9e-05 | [-.002, .002] |
| sta lib x agy | 4e-04 | [-.003, .004] |  | .002 | [-.002, .005] |  | .005 | [.002, .008] |  | lib x agr | .005 | [.003, .006] |
| lib x sta lib x com | .003 | [.001, .004] |  | .001 | [-3e-04, .003] |  | 2e-04 | [-.002, .002] |  | sta lib x agr | -.009 | [-.012, -.007] |
| lib x sta lib x agy | -.004 | [-.006, -.002] |  | -.002 | [-.004, -2e-04] |  | -.001 | [-.003, .001] |  | lib x cns | .002 | [.001, .003] |
|  |  |  |  |  |  |  |  |  |  | sta lib x cns | .001 | [-.002, .004] |
|  |  |  |  |  |  |  |  |  |  | lib x opn | -.002 | [-.003, -4e-04] |
|  |  |  |  |  |  |  |  |  |  | sta lib x opn | .007 | [.004, .009] |
|  |  |  |  |  |  |  |  |  |  | lib x ext | -.002 | [-.003, -4e-04] |
|  |  |  |  |  |  |  |  |  |  | sta lib x ext | -.002 | [-.005, .001] |
|  |  |  |  |  |  |  |  |  |  | lib x neu | .004 | [.002, .005] |
|  |  |  |  |  |  |  |  |  |  | sta lib x neu | -2e-04 | [-.005, .004] |
|  |  |  |  |  |  |  |  |  |  | lib x sta lib x agr | .001 | [-.001, .002] |
|  |  |  |  |  |  |  |  |  |  | lib x sta lib x cns | -.001 | [-.003, 2e-04] |
|  |  |  |  |  |  |  |  |  |  | lib x sta lib x opn | .001 | [-.001, .002] |
|  |  |  |  |  |  |  |  |  |  | lib x sta lib x ext | -.003 | [-.005, -.002] |
|  |  |  |  |  |  |  |  |  |  | lib x sta lib x neu | -.002 | [-.004, -7e-05] |

Note: TS = target scale, AC = ant colony, BF = brute force, lib = liberalism, sta lib = state-level liberalism, agy = agency, com = communion, agr = agreeableness, cns = conscientiousness, ext = extraversion, opn = openness, neu = neuroticism.


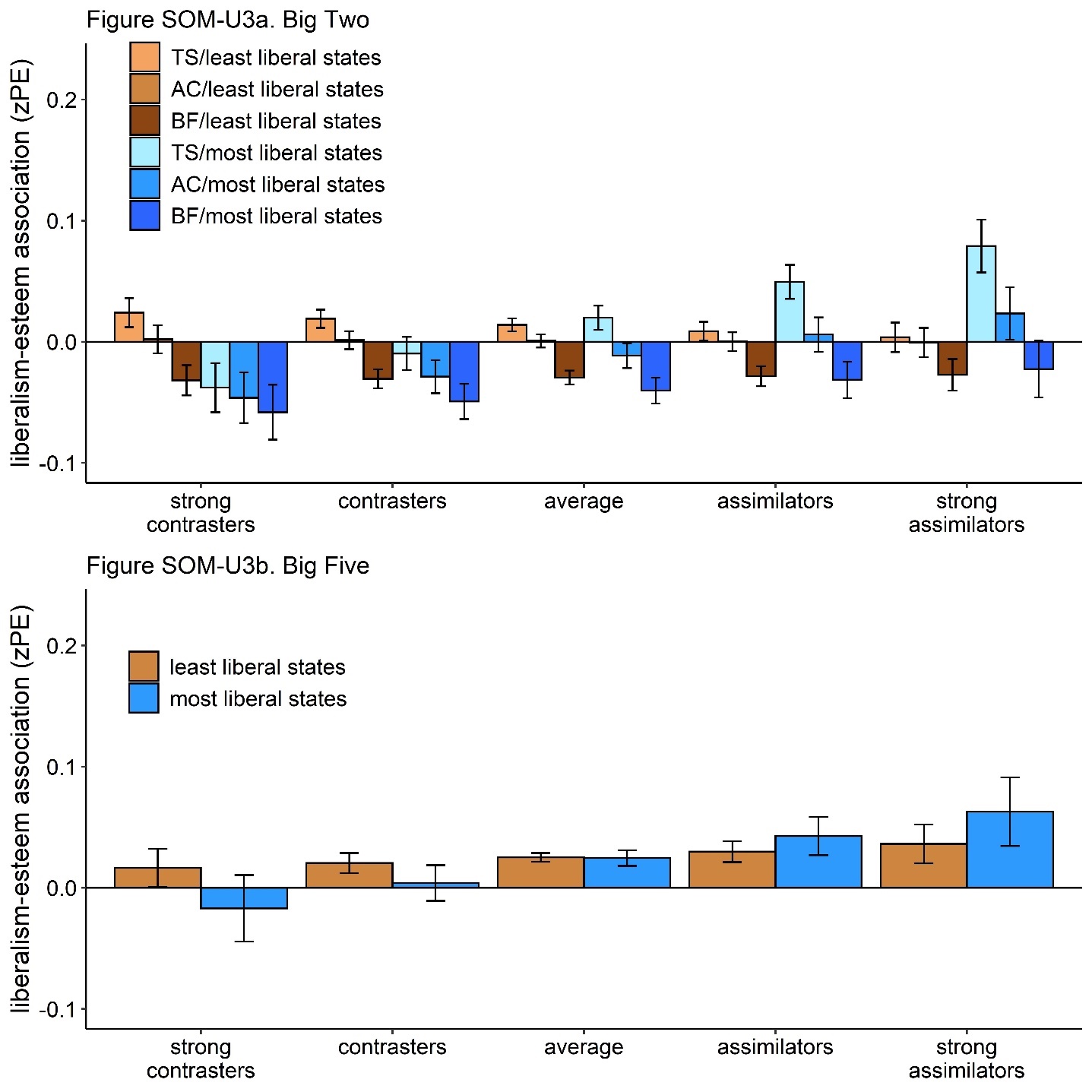


**Fig. S3.** Person-culture match effect for assimilators and contrasters with liberalism as the match domain. (Strong) assimilators = mean + 1 SD (+ 2 SD) on all assimilation-eliciting traits and mean - 1 SD (-2 SD) on all contrast-eliciting traits, (strong) contrasters = mean + 1 SD (+ 2 SD) on all contrast-eliciting traits and mean - 1 SD (-2 SD) on all assimilation-eliciting traits, TS = target scale, AC = ant colony, BF = brute force. Error bars indicate 95% confidence intervals.

**Study S4.** The main text focuses on self-esteem as the well-being indicator. Self-esteem is ideal because most relevant theories consider self-esteem to be the most immediate beneficiary of person-culture match (for a review, see Gebauer et al., 2015). Other well-being benefits, in turn, are considered indirect, driven by self-esteem (Pyszczynski et al., 2004; Rosenberg, 1965). Nonetheless, we sought to test for the generalizability of our main-text results to other well-being indicators. For that purpose, the GPIPP includes one particularly suitable construct: depression (“I see myself as someone who is depressed, blue;” 1 = *strongly disagree*, 5 = *strongly agree*).^S4^

To extract the relevant GPIPP data, we applied the main-text selection criteria, while replacing the main text’s single-item self-esteem scale with the single-item depression scale. The resultant sample contained data from 2,672,742 participants across 102 countries (62.18% women, *M*_age_ = 25.55 years, *SD*_age_ = 10.91; questionnaire language: 66.50% English, 19.33% Spanish, 8.21% German, 5.96% Dutch). The present sample was virtually identical to the main-text sample and that explains why the psychometric properties were virtually identical, too.

We used the exact same statistical modeling strategy as that used in the main text. Table S4 includes the results of the four models (three Big Two models, Big Five model).^S5^ The table reveals that basic personality traits again moderated person-culture match’s well-being benefits. Specifically, for the most part, the results replicated the main-text results. Notably, five three-way interactions that were significant with self-esteem as the criterion did not reach significance with depression as the criterion. This is not that surprising when one considers that self-esteem is typically regarded as the most immediate beneficiary of person-culture match, whereas other well-being indicators are regarded as more indirect and driven by self-esteem (for a review, see Gebauer et al., 2015). Importantly, though, Fig. S4 shows that the overall result pattern was very similar to the main text’s pattern (Fig. 1).

**Table S4.** The Person-Culture Match Effect on Depression Moderated by the Big Two and the Big Five

| **Big Two** | | | | | | | | |  | **Big Five** | | |
| --- | --- | --- | --- | --- | --- | --- | --- | --- | --- | --- | --- | --- |
|  | **TS** | |  | **AC** | |  | **BF** | |  |  |  |  |
|  | ***zPE*** | **95% CI** |  | ***zPE*** | **95% CI** |  | ***zPE*** | **95% CI** |  |  | ***zPE*** | **95% CI** |
| (intercept) | .066 | [.034, .098] |  | .066 | [.034, .098] |  | .067 | [.035, .099] |  | (intercept) | .064 | [.032, .096] |
| rel | .010 | [.004, .016] |  | -.004 | [-.009, .002] |  | .004 | [-.002, .010] |  | rel | 9e-05 | [-.004, .004] |
| cnt rel | .014 | [-.018, .046] |  | .013 | [-.019, .045] |  | .014 | [-.018, .046] |  | cnt rel | .012 | [-.020, .045] |
| com | -.204 | [-.211, -.197] |  | -.194 | [-.203, -.185] |  | -.195 | [-.204, -.186] |  | agr | -.059 | [-.068, -.049] |
| agy | -.311 | [-.319, -.304] |  | -.318 | [-.326, -.310] |  | -.229 | [-.237, -.222] |  | cns | -.076 | [-.083, -.070] |
| rel x cnt rel | -.017 | [-.023, -.011] |  | -.018 | [-.024, -.012] |  | -.022 | [-.028, -.015] |  | opn | .100 | [.094, .105] |
| rel x com | -.011 | [-.012, -.010] |  | -.006 | [-.007, -.005] |  | -.007 | [-.009, -.006] |  | ext | -.241 | [-.250, -.231] |
| cnt rel x com | -.005 | [-.011, .002] |  | .007 | [-.002, .016] |  | .003 | [-.006, .012] |  | neu | .435 | [.422, .448] |
| rel x agy | .011 | [.010, .012] |  | .011 | [.010, .012] |  | .006 | [.004, .007] |  | rel x cnt rel | -.010 | [-.014, -.006] |
| cnt rel x agy | .025 | [.017, .032] |  | .017 | [.009, .025] |  | .004 | [-.003, .012] |  | rel x agr | -.004 | [-.005, -.003] |
| rel x cnt rel x com | -.003 | [-.005, -.002] |  | -.003 | [-.004, -.001] |  | -.005 | [-.006, -.003] |  | cnt rel x agr | .001 | [-.008, .010] |
| rel x cnt rel x agy | .001 | [-3e-04, .003] |  | .002 | [.001, .004] |  | .002 | [-7e-05, .003] |  | rel x cns | .003 | [.002, .004] |
|  |  |  |  |  |  |  |  |  |  | cnt rel x cns | -.007 | [-.013, -3e-04] |
|  |  |  |  |  |  |  |  |  |  | rel x opn | -.003 | [-.004, -.002] |
|  |  |  |  |  |  |  |  |  |  | cnt rel x opn | -.007 | [-.012, -.002] |
|  |  |  |  |  |  |  |  |  |  | rel x ext | .009 | [.008, .010] |
|  |  |  |  |  |  |  |  |  |  | cnt rel x ext | .026 | [.016, .036] |
|  |  |  |  |  |  |  |  |  |  | rel x neu | -.006 | [-.007, -.005] |
|  |  |  |  |  |  |  |  |  |  | cnt rel x neu | .009 | [-.004, .022] |
|  |  |  |  |  |  |  |  |  |  | rel x cnt rel x agr | -.001 | [-.002, .001] |
|  |  |  |  |  |  |  |  |  |  | rel x cnt rel x cns | -2e-04 | [-.002, .001] |
|  |  |  |  |  |  |  |  |  |  | rel x cnt rel x opn | 4e-04 | [-.001, .002] |
|  |  |  |  |  |  |  |  |  |  | rel x cnt rel x ext | .002 | [5e-04, .003] |
|  |  |  |  |  |  |  |  |  |  | rel x cnt rel x neu | -.005 | [-.006, -.003] |

Note: TS = target scale, AC = ant colony, BF = brute force, rel = religiosity, cnt rel = country-level religiosity, agy = agency, com = communion, agr = agreeableness, cns = conscientiousness, ext = extraversion, opn = openness, neu = neuroticism.


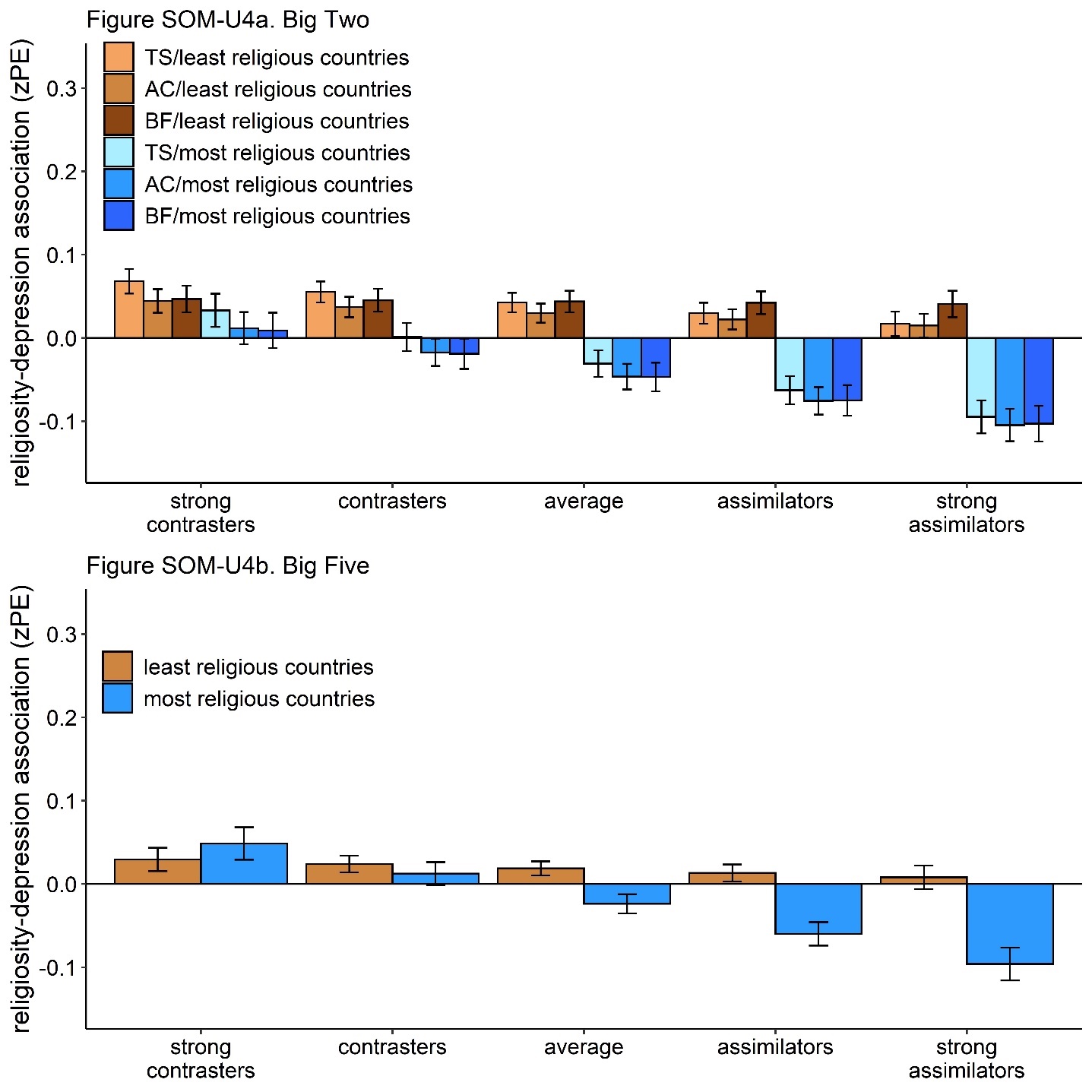


**Fig. S4.** Person-culture match effect for assimilators and contrasters with depression as the well-being indicator. (Strong) assimilators = mean + 1 SD (+ 2 SD) on all assimilation-eliciting traits and mean - 1 SD (-2 SD) on all contrast-eliciting traits, (strong) contrasters = mean + 1 SD (+ 2 SD) on all contrast-eliciting traits and mean - 1 SD (-2 SD) on all assimilation-eliciting traits, TS = target scale, AC = ant colony, BF = brute force. Error bars indicate 95% confidence intervals.

**Section S1.** The full GPIPP dataset underwent six steps of data-cleaning prior to making it available to researchers: Participants were excluded if they indicated that they (1) currently live in a U.S. state and also in a country outside the U.S., (2) do not want their data to be used for scientific purposes, (3) have completed the same study before, or (4) did not respond truthfully. In addition, (5) one GPIPP study was excluded because its mean Big Five scores differed vastly from the scores of all other GPIPP studies and (6) random responders were also excluded (identified with three complementary methods: within-participant correlation, Mahalanobis distances, and maximal long-string; Meade & Craig, 2012).

**Section S2.** Table S5 lists all 102 countries analyzed in the main text, including their sample size and demographic composition (age, sex). The table also includes each country’s average religiosity (i.e., our country-level religiosity score). The countries are sorted according to their country-level religiosity, starting with the most religious country.

**Table S5.** List of the 102 Countries From the Main Text, Including Their Demographics (Age, Sex) and Their Average Religiosity

| **Country** | ***N*** | **Age** | | **% Women** | **Country-Level** |
| --- | --- | --- | --- | --- | --- |
|  |  | ***M*** | ***SD*** |  | **Religiosity** |
| Nigeria | 586 | 28.04 | 8.11 | 50.09 | 3.70 |
| Kenya | 626 | 26.97 | 8.71 | 61.18 | 3.50 |
| Jamaica | 798 | 25.88 | 9.54 | 75.94 | 3.44 |
| Sri Lanka | 449 | 26.32 | 9.77 | 54.36 | 3.34 |
| Pakistan | 3,738 | 23.18 | 6.81 | 53.35 | 3.33 |
| Philippines | 17,589 | 21.48 | 6.73 | 74.72 | 3.28 |
| Egypt | 2,068 | 23.48 | 7.71 | 66.91 | 3.24 |
| Suriname | 386 | 33.21 | 11.10 | 70.65 | 3.18 |
| Indonesia | 2,649 | 24.50 | 7.87 | 57.10 | 3.17 |
| Saudi Arabia | 1,107 | 25.71 | 9.61 | 52.72 | 3.17 |
| Dominican Republic | 5,523 | 23.79 | 8.22 | 73.89 | 3.16 |
| India | 19,350 | 24.84 | 7.21 | 47.52 | 3.14 |
| Panama | 2,292 | 25.92 | 9.99 | 68.48 | 3.13 |
| Qatar | 363 | 26.74 | 10.06 | 63.64 | 3.10 |
| Bahamas | 575 | 24.74 | 12.26 | 69.54 | 3.08 |
| El Salvador | 3,335 | 24.62 | 8.68 | 64.87 | 3.08 |
| Kuwait | 519 | 25.19 | 9.51 | 63.25 | 3.08 |
| Trinidad and Tobago | 690 | 25.40 | 9.23 | 66.91 | 3.08 |
| Puerto Rico | 3,882 | 27.15 | 10.62 | 67.29 | 3.07 |
| Mauritius | 407 | 24.03 | 7.43 | 56.19 | 3.06 |
| Honduras | 2,728 | 25.18 | 8.39 | 68.40 | 3.05 |
| Jordan | 509 | 24.65 | 8.58 | 62.48 | 3.05 |
| Nicaragua | 2,070 | 25.58 | 9.08 | 71.00 | 3.04 |
| Malaysia | 8,355 | 23.36 | 7.53 | 65.57 | 3.02 |
| Morocco | 328 | 24.21 | 9.40 | 66.36 | 2.99 |
| United Arab Emirates | 3,888 | 26.92 | 10.87 | 58.01 | 2.97 |
| Paraguay | 3,296 | 23.92 | 8.85 | 69.67 | 2.97 |
| South Africa | 6,202 | 28.21 | 10.57 | 64.88 | 2.97 |
| Guatemala | 5,101 | 24.66 | 8.37 | 64.53 | 2.95 |
| Ecuador | 8,325 | 25.89 | 9.87 | 64.54 | 2.94 |
| Lebanon | 1,074 | 23.60 | 7.35 | 60.19 | 2.93 |
| Venezuela | 18,448 | 23.58 | 9.42 | 71.35 | 2.93 |
| Bangladesh | 610 | 24.64 | 12.83 | 46.20 | 2.89 |
| Colombia | 32,875 | 23.54 | 8.69 | 68.32 | 2.89 |
| Barbados | 366 | 28.31 | 12.77 | 66.30 | 2.88 |
| Bolivia | 5,828 | 23.47 | 7.62 | 64.69 | 2.86 |
| Peru | 22,027 | 24.78 | 9.25 | 63.71 | 2.86 |
| Bosnia-Herzegovina | 383 | 26.09 | 10.67 | 63.25 | 2.84 |
| Bahrain | 424 | 24.67 | 10.63 | 64.85 | 2.83 |
| CostaRica | 5,755 | 24.34 | 8.62 | 67.85 | 2.79 |
| Thailand | 1,959 | 25.89 | 10.15 | 60.05 | 2.74 |
| United States | 1,247,498 | 25.31 | 11.24 | 61.84 | 2.70 |
| Netherlands Antilles | 1,917 | 30.36 | 12.50 | 72.25 | 2.67 |
| Afghanistan | 482 | 31.15 | 18.24 | 45.70 | 2.65 |
| Albania | 493 | 25.74 | 12.83 | 61.54 | 2.64 |
| Singapore | 11,832 | 21.87 | 7.54 | 63.19 | 2.62 |
| Chile | 42,849 | 24.84 | 10.52 | 68.96 | 2.61 |
| Aruba | 687 | 29.29 | 13.32 | 70.66 | 2.58 |
| Brasil | 3,658 | 29.43 | 11.12 | 42.95 | 2.56 |
| Cyprus | 796 | 26.93 | 10.57 | 64.98 | 2.56 |
| Belize | 370 | 26.50 | 14.01 | 62.30 | 2.55 |
| Taiwan | 1,217 | 26.41 | 9.52 | 60.92 | 2.54 |
| Mexico | 128,080 | 23.27 | 8.16 | 63.25 | 2.53 |
| Romania | 3,099 | 23.53 | 7.59 | 63.55 | 2.50 |
| Vietnam | 524 | 26.05 | 9.85 | 59.08 | 2.48 |
| Iran | 995 | 25.50 | 7.57 | 57.80 | 2.45 |
| Bermuda | 303 | 29.65 | 16.35 | 60.13 | 2.44 |
| Armenia | 757 | 25.28 | 12.14 | 71.85 | 2.42 |
| South Korea | 2,317 | 27.41 | 8.24 | 43.02 | 2.42 |
| Turkey | 1,510 | 26.07 | 8.86 | 54.86 | 2.39 |
| Argentina | 85,415 | 24.14 | 9.29 | 71.36 | 2.38 |
| China | 6,652 | 27.47 | 8.03 | 61.34 | 2.37 |
| Croatia | 1,697 | 23.74 | 7.21 | 60.47 | 2.36 |
| Hong Kong | 4,270 | 25.76 | 9.39 | 65.64 | 2.36 |
| Malta | 423 | 25.78 | 10.25 | 59.13 | 2.36 |
| Cuba | 935 | 27.73 | 10.49 | 73.15 | 2.34 |
| Serbia-Montenegro | 1,437 | 24.44 | 7.37 | 58.59 | 2.30 |
| Russia | 1,221 | 25.30 | 8.72 | 63.70 | 2.28 |
| Slovak Republic | 371 | 24.90 | 9.35 | 57.45 | 2.27 |
| Italy | 5,055 | 29.03 | 10.72 | 58.00 | 2.26 |
| Bulgaria | 913 | 23.34 | 8.85 | 63.11 | 2.24 |
| Japan | 3,956 | 26.70 | 9.96 | 56.85 | 2.24 |
| Greece | 2,829 | 26.14 | 9.03 | 62.93 | 2.23 |
| Ireland | 11,942 | 23.87 | 8.75 | 57.52 | 2.21 |
| Uruguay | 5,938 | 25.04 | 10.54 | 72.72 | 2.19 |
| Canada | 117,059 | 24.29 | 10.78 | 60.07 | 2.18 |
| Poland | 2,522 | 24.15 | 7.86 | 52.81 | 2.18 |
| Hungary | 1,339 | 26.37 | 9.37 | 60.02 | 2.12 |
| New Zealand | 14,688 | 26.57 | 12.06 | 63.61 | 2.12 |
| Australia | 52,925 | 25.34 | 11.20 | 59.51 | 2.11 |
| Czech Republic | 868 | 25.19 | 9.12 | 45.72 | 2.09 |
| Austria | 23,879 | 27.25 | 11.58 | 60.88 | 2.08 |
| Iceland | 699 | 25.97 | 9.68 | 51.72 | 2.08 |
| Switzerland | 31,487 | 28.26 | 12.50 | 61.49 | 2.05 |
| Germany | 167,780 | 29.36 | 11.94 | 58.96 | 2.01 |
| Latvia | 473 | 23.93 | 7.10 | 61.78 | 2.01 |
| Israel | 2,446 | 27.49 | 10.54 | 55.52 | 2.00 |
| Lithuania | 453 | 23.77 | 8.10 | 63.11 | 1.98 |
| Portugal | 2,063 | 25.81 | 9.62 | 51.61 | 1.98 |
| Finland | 7,838 | 24.41 | 8.70 | 60.57 | 1.94 |
| France | 5,702 | 28.11 | 10.81 | 56.80 | 1.94 |
| Netherlands | 138,974 | 29.19 | 12.21 | 62.73 | 1.93 |
| Andorra | 401 | 26.13 | 11.63 | 62.66 | 1.92 |
| United Kingdom | 126,659 | 24.83 | 10.59 | 55.20 | 1.90 |
| Spain | 128,119 | 24.19 | 9.30 | 68.30 | 1.90 |
| Luxembourg | 666 | 28.47 | 12.24 | 61.63 | 1.87 |
| Slovenia | 721 | 24.60 | 8.73 | 55.17 | 1.84 |
| Estonia | 721 | 22.71 | 8.86 | 63.28 | 1.82 |
| Denmark | 5,054 | 27.69 | 10.31 | 47.94 | 1.75 |
| Belgium | 34,886 | 27.00 | 11.54 | 65.20 | 1.73 |
| Norway | 12,333 | 27.71 | 10.53 | 56.64 | 1.69 |
| Sweden | 12,144 | 27.55 | 10.79 | 55.33 | 1.67 |

**Section S3.** Table S6 includes means and standard deviations of each main-text measure for each country analyzed in the main text.

**Table S6.** Mean (Standard Deviation) of Each Main-Text Measure for Each Country Analyzed in the Main Text

| **Country** |  | **Self-esteem** |  | **Religiosity** |  | **Big Two** | | | | | | | |  | **Big Five** | | | | |
| --- | --- | --- | --- | --- | --- | --- | --- | --- | --- | --- | --- | --- | --- | --- | --- | --- | --- | --- | --- |
|  |  |  |  |  |  | **TS** | |  | **AC** | |  | **BF** | |  |  |  |  |  |  |
|  |  |  |  |  |  | **agy** | **com** |  | **agy** | **com** |  | **agy** | **com** |  | **agr** | **cns** | **opn** | **ext** | **neu** |
| Afghanistan |  | 3.44 (1.26) |  | 2.65 (1.41) |  | 3.40 (0.80) | 3.53 (0.73) |  | 3.47 (0.69) | 3.51 (0.71) |  | 3.45 (0.66) | 3.53 (0.73) |  | 3.45 (0.73) | 3.39 (0.81) | 3.28 (0.83) | 3.70 (0.72) | 2.92 (0.85) |
| Albania |  | 3.41 (1.19) |  | 2.64 (1.42) |  | 3.42 (0.79) | 3.63 (0.65) |  | 3.50 (0.63) | 3.61 (0.65) |  | 3.43 (0.56) | 3.60 (0.66) |  | 3.57 (0.67) | 3.42 (0.74) | 3.28 (0.84) | 3.78 (0.63) | 3.01 (0.79) |
| Andorra |  | 3.17 (1.27) |  | 1.92 (1.20) |  | 3.39 (0.85) | 3.67 (0.60) |  | 3.48 (0.67) | 3.61 (0.57) |  | 3.39 (0.57) | 3.65 (0.59) |  | 3.52 (0.61) | 3.33 (0.71) | 3.26 (0.88) | 3.77 (0.63) | 3.05 (0.84) |
| Argentina |  | 3.05 (1.30) |  | 2.38 (1.31) |  | 3.38 (0.80) | 3.58 (0.62) |  | 3.46 (0.65) | 3.52 (0.61) |  | 3.34 (0.57) | 3.56 (0.63) |  | 3.41 (0.63) | 3.23 (0.72) | 3.23 (0.82) | 3.79 (0.65) | 3.33 (0.83) |
| Armenia |  | 3.13 (1.27) |  | 2.42 (1.30) |  | 3.42 (0.81) | 3.61 (0.60) |  | 3.47 (0.66) | 3.53 (0.61) |  | 3.37 (0.59) | 3.58 (0.61) |  | 3.46 (0.62) | 3.29 (0.73) | 3.28 (0.84) | 3.78 (0.63) | 3.27 (0.84) |
| Aruba |  | 3.30 (1.29) |  | 2.58 (1.35) |  | 3.49 (0.75) | 3.63 (0.65) |  | 3.55 (0.63) | 3.59 (0.64) |  | 3.43 (0.56) | 3.65 (0.65) |  | 3.50 (0.67) | 3.45 (0.75) | 3.34 (0.78) | 3.79 (0.63) | 3.09 (0.86) |
| Australia |  | 3.22 (1.21) |  | 2.11 (1.32) |  | 3.37 (0.78) | 3.65 (0.65) |  | 3.40 (0.62) | 3.64 (0.65) |  | 3.40 (0.56) | 3.70 (0.66) |  | 3.62 (0.68) | 3.37 (0.74) | 3.27 (0.83) | 3.72 (0.63) | 2.95 (0.83) |
| Austria |  | 3.38 (1.16) |  | 2.08 (1.26) |  | 3.51 (0.80) | 3.56 (0.62) |  | 3.37 (0.65) | 3.57 (0.64) |  | 3.56 (0.56) | 3.53 (0.62) |  | 3.44 (0.66) | 3.46 (0.82) | 3.44 (0.86) | 3.76 (0.68) | 2.95 (0.87) |
| Bahamas |  | 3.66 (1.16) |  | 3.08 (1.43) |  | 3.40 (0.76) | 3.73 (0.67) |  | 3.45 (0.60) | 3.72 (0.64) |  | 3.45 (0.57) | 3.73 (0.67) |  | 3.65 (0.69) | 3.54 (0.74) | 3.26 (0.80) | 3.77 (0.63) | 2.90 (0.81) |
| Bahrain |  | 3.46 (1.24) |  | 2.83 (1.32) |  | 3.33 (0.77) | 3.65 (0.70) |  | 3.47 (0.64) | 3.60 (0.68) |  | 3.41 (0.54) | 3.65 (0.67) |  | 3.59 (0.70) | 3.32 (0.72) | 3.20 (0.79) | 3.76 (0.59) | 3.14 (0.90) |
| Bangladesh |  | 3.59 (1.21) |  | 2.89 (1.30) |  | 3.28 (0.78) | 3.61 (0.68) |  | 3.37 (0.64) | 3.60 (0.66) |  | 3.39 (0.59) | 3.57 (0.64) |  | 3.56 (0.66) | 3.26 (0.75) | 3.16 (0.84) | 3.68 (0.64) | 3.04 (0.87) |
| Barbados |  | 3.35 (1.24) |  | 2.88 (1.45) |  | 3.30 (0.76) | 3.64 (0.69) |  | 3.43 (0.63) | 3.58 (0.69) |  | 3.35 (0.59) | 3.65 (0.70) |  | 3.54 (0.73) | 3.48 (0.79) | 3.18 (0.81) | 3.72 (0.67) | 3.05 (0.89) |
| Belgium |  | 2.94 (1.13) |  | 1.73 (1.07) |  | 3.49 (0.73) | 3.63 (0.60) |  | 3.45 (0.62) | 3.54 (0.61) |  | 3.42 (0.55) | 3.69 (0.62) |  | 3.57 (0.63) | 3.36 (0.76) | 3.43 (0.78) | 3.61 (0.66) | 3.02 (0.83) |
| Belize |  | 3.33 (1.21) |  | 2.55 (1.35) |  | 3.35 (0.80) | 3.63 (0.65) |  | 3.42 (0.64) | 3.58 (0.67) |  | 3.38 (0.59) | 3.63 (0.66) |  | 3.56 (0.67) | 3.38 (0.75) | 3.22 (0.85) | 3.70 (0.63) | 3.05 (0.83) |
| Bermuda |  | 3.43 (1.19) |  | 2.44 (1.41) |  | 3.46 (0.79) | 3.59 (0.71) |  | 3.47 (0.62) | 3.57 (0.69) |  | 3.43 (0.58) | 3.63 (0.72) |  | 3.54 (0.71) | 3.42 (0.75) | 3.35 (0.84) | 3.71 (0.68) | 2.93 (0.81) |
| Bolivia |  | 3.38 (1.23) |  | 2.86 (1.28) |  | 3.33 (0.78) | 3.57 (0.62) |  | 3.51 (0.64) | 3.51 (0.61) |  | 3.39 (0.56) | 3.48 (0.62) |  | 3.38 (0.62) | 3.27 (0.69) | 3.14 (0.80) | 3.88 (0.61) | 3.21 (0.79) |
| Bosnia-Herzegovina |  | 3.60 (1.16) |  | 2.84 (1.41) |  | 3.52 (0.73) | 3.63 (0.58) |  | 3.56 (0.56) | 3.58 (0.58) |  | 3.60 (0.53) | 3.59 (0.59) |  | 3.55 (0.62) | 3.47 (0.69) | 3.43 (0.77) | 3.85 (0.55) | 3.00 (0.82) |
| Brasil |  | 3.39 (1.18) |  | 2.56 (1.41) |  | 3.35 (0.75) | 3.63 (0.61) |  | 3.52 (0.59) | 3.62 (0.60) |  | 3.38 (0.53) | 3.60 (0.62) |  | 3.55 (0.63) | 3.45 (0.70) | 3.16 (0.79) | 3.83 (0.60) | 2.94 (0.81) |
| Bulgaria |  | 3.37 (1.21) |  | 2.24 (1.29) |  | 3.41 (0.76) | 3.58 (0.64) |  | 3.52 (0.60) | 3.47 (0.62) |  | 3.51 (0.56) | 3.54 (0.64) |  | 3.49 (0.67) | 3.28 (0.75) | 3.28 (0.78) | 3.91 (0.55) | 3.07 (0.83) |
| Canada |  | 3.31 (1.19) |  | 2.18 (1.34) |  | 3.38 (0.78) | 3.69 (0.65) |  | 3.42 (0.60) | 3.68 (0.64) |  | 3.39 (0.56) | 3.74 (0.66) |  | 3.65 (0.68) | 3.42 (0.72) | 3.27 (0.82) | 3.71 (0.64) | 2.95 (0.82) |
| Chile |  | 3.34 (1.29) |  | 2.61 (1.35) |  | 3.42 (0.82) | 3.67 (0.62) |  | 3.62 (0.66) | 3.59 (0.61) |  | 3.47 (0.57) | 3.65 (0.65) |  | 3.46 (0.64) | 3.47 (0.75) | 3.23 (0.83) | 3.93 (0.62) | 3.06 (0.83) |
| China |  | 3.62 (1.15) |  | 2.37 (1.24) |  | 3.19 (0.69) | 3.70 (0.55) |  | 3.27 (0.61) | 3.70 (0.55) |  | 3.28 (0.55) | 3.72 (0.56) |  | 3.68 (0.57) | 3.46 (0.66) | 3.09 (0.70) | 3.52 (0.63) | 2.86 (0.74) |
| Colombia |  | 3.64 (1.22) |  | 2.89 (1.36) |  | 3.46 (0.81) | 3.71 (0.63) |  | 3.64 (0.65) | 3.61 (0.61) |  | 3.46 (0.56) | 3.64 (0.63) |  | 3.50 (0.63) | 3.47 (0.72) | 3.25 (0.81) | 3.95 (0.60) | 3.05 (0.83) |
| CostaRica |  | 3.53 (1.23) |  | 2.79 (1.32) |  | 3.45 (0.79) | 3.68 (0.62) |  | 3.64 (0.64) | 3.59 (0.60) |  | 3.46 (0.54) | 3.64 (0.62) |  | 3.46 (0.62) | 3.46 (0.70) | 3.25 (0.81) | 3.92 (0.62) | 3.04 (0.82) |
| Croatia |  | 3.32 (1.17) |  | 2.36 (1.35) |  | 3.40 (0.74) | 3.56 (0.61) |  | 3.48 (0.58) | 3.50 (0.61) |  | 3.52 (0.52) | 3.54 (0.64) |  | 3.51 (0.65) | 3.32 (0.71) | 3.30 (0.79) | 3.83 (0.53) | 2.97 (0.84) |
| Cuba |  | 3.70 (1.24) |  | 2.34 (1.31) |  | 3.58 (0.81) | 3.86 (0.59) |  | 3.71 (0.66) | 3.73 (0.59) |  | 3.50 (0.55) | 3.83 (0.61) |  | 3.64 (0.61) | 3.46 (0.69) | 3.36 (0.81) | 4.02 (0.58) | 3.11 (0.84) |
| Cyprus |  | 3.29 (1.25) |  | 2.56 (1.31) |  | 3.37 (0.79) | 3.68 (0.60) |  | 3.47 (0.64) | 3.64 (0.59) |  | 3.42 (0.55) | 3.69 (0.62) |  | 3.60 (0.62) | 3.47 (0.70) | 3.22 (0.80) | 3.79 (0.60) | 3.09 (0.79) |
| Czech Republic |  | 3.37 (1.18) |  | 2.09 (1.31) |  | 3.30 (0.75) | 3.53 (0.63) |  | 3.46 (0.61) | 3.47 (0.64) |  | 3.49 (0.52) | 3.51 (0.64) |  | 3.43 (0.67) | 3.33 (0.74) | 3.18 (0.81) | 3.87 (0.56) | 2.91 (0.83) |
| Denmark |  | 3.43 (1.14) |  | 1.75 (1.14) |  | 3.43 (0.70) | 3.65 (0.61) |  | 3.56 (0.57) | 3.66 (0.62) |  | 3.52 (0.53) | 3.68 (0.63) |  | 3.62 (0.65) | 3.45 (0.72) | 3.34 (0.76) | 3.80 (0.60) | 2.73 (0.79) |
| Dominican Republic |  | 3.55 (1.22) |  | 3.16 (1.30) |  | 3.40 (0.78) | 3.87 (0.60) |  | 3.61 (0.65) | 3.76 (0.59) |  | 3.47 (0.56) | 3.73 (0.60) |  | 3.65 (0.61) | 3.53 (0.69) | 3.17 (0.80) | 3.96 (0.60) | 3.04 (0.84) |
| Ecuador |  | 3.66 (1.21) |  | 2.94 (1.33) |  | 3.42 (0.80) | 3.79 (0.64) |  | 3.64 (0.68) | 3.72 (0.61) |  | 3.48 (0.59) | 3.71 (0.63) |  | 3.58 (0.62) | 3.52 (0.74) | 3.21 (0.80) | 3.93 (0.61) | 2.99 (0.84) |
| Egypt |  | 3.50 (1.23) |  | 3.24 (1.14) |  | 3.31 (0.69) | 3.82 (0.61) |  | 3.48 (0.60) | 3.73 (0.60) |  | 3.34 (0.54) | 3.81 (0.60) |  | 3.75 (0.62) | 3.40 (0.71) | 3.19 (0.69) | 3.74 (0.57) | 3.27 (0.84) |
| El Salvador |  | 3.58 (1.22) |  | 3.08 (1.33) |  | 3.42 (0.81) | 3.71 (0.65) |  | 3.64 (0.67) | 3.62 (0.61) |  | 3.49 (0.56) | 3.63 (0.64) |  | 3.49 (0.63) | 3.46 (0.73) | 3.22 (0.82) | 3.94 (0.61) | 3.07 (0.86) |
| Estonia |  | 3.28 (1.23) |  | 1.82 (1.17) |  | 3.19 (0.76) | 3.44 (0.62) |  | 3.44 (0.60) | 3.37 (0.63) |  | 3.35 (0.53) | 3.42 (0.65) |  | 3.37 (0.67) | 3.15 (0.70) | 3.05 (0.79) | 3.85 (0.58) | 3.10 (0.84) |
| Finland |  | 3.26 (1.17) |  | 1.94 (1.20) |  | 3.21 (0.72) | 3.53 (0.61) |  | 3.34 (0.56) | 3.53 (0.61) |  | 3.41 (0.52) | 3.56 (0.63) |  | 3.50 (0.65) | 3.27 (0.68) | 3.10 (0.76) | 3.75 (0.57) | 3.01 (0.79) |
| France |  | 3.15 (1.22) |  | 1.94 (1.23) |  | 3.36 (0.73) | 3.64 (0.60) |  | 3.51 (0.60) | 3.62 (0.61) |  | 3.46 (0.55) | 3.61 (0.61) |  | 3.55 (0.63) | 3.39 (0.72) | 3.24 (0.80) | 3.88 (0.62) | 2.95 (0.84) |
| Germany |  | 3.28 (1.15) |  | 2.01 (1.25) |  | 3.45 (0.79) | 3.54 (0.59) |  | 3.33 (0.64) | 3.54 (0.62) |  | 3.54 (0.55) | 3.51 (0.61) |  | 3.42 (0.64) | 3.44 (0.77) | 3.37 (0.85) | 3.76 (0.65) | 3.04 (0.86) |
| Greece |  | 3.21 (1.23) |  | 2.23 (1.29) |  | 3.33 (0.72) | 3.62 (0.63) |  | 3.48 (0.58) | 3.58 (0.62) |  | 3.48 (0.53) | 3.61 (0.63) |  | 3.60 (0.65) | 3.29 (0.73) | 3.22 (0.74) | 3.90 (0.57) | 3.17 (0.81) |
| Guatemala |  | 3.50 (1.23) |  | 2.95 (1.30) |  | 3.35 (0.80) | 3.70 (0.62) |  | 3.57 (0.66) | 3.59 (0.60) |  | 3.42 (0.58) | 3.58 (0.63) |  | 3.47 (0.62) | 3.40 (0.69) | 3.14 (0.81) | 3.89 (0.62) | 3.11 (0.82) |
| Honduras |  | 3.59 (1.25) |  | 3.05 (1.32) |  | 3.36 (0.81) | 3.76 (0.64) |  | 3.63 (0.66) | 3.65 (0.62) |  | 3.46 (0.57) | 3.64 (0.63) |  | 3.51 (0.63) | 3.51 (0.71) | 3.14 (0.82) | 3.94 (0.61) | 3.06 (0.84) |
| Hong Kong |  | 3.42 (1.06) |  | 2.36 (1.22) |  | 3.20 (0.70) | 3.60 (0.57) |  | 3.21 (0.61) | 3.61 (0.56) |  | 3.21 (0.55) | 3.59 (0.57) |  | 3.57 (0.58) | 3.28 (0.68) | 3.13 (0.71) | 3.41 (0.62) | 3.04 (0.78) |
| Hungary |  | 3.34 (1.16) |  | 2.12 (1.25) |  | 3.37 (0.76) | 3.56 (0.65) |  | 3.41 (0.62) | 3.53 (0.65) |  | 3.51 (0.54) | 3.57 (0.66) |  | 3.49 (0.69) | 3.45 (0.72) | 3.26 (0.80) | 3.85 (0.57) | 2.98 (0.83) |
| Iceland |  | 3.24 (1.19) |  | 2.08 (1.32) |  | 3.29 (0.75) | 3.66 (0.63) |  | 3.46 (0.62) | 3.66 (0.61) |  | 3.45 (0.55) | 3.65 (0.65) |  | 3.61 (0.66) | 3.35 (0.77) | 3.18 (0.79) | 3.79 (0.63) | 2.83 (0.86) |
| India |  | 3.89 (1.08) |  | 3.14 (1.32) |  | 3.38 (0.70) | 3.80 (0.60) |  | 3.46 (0.59) | 3.78 (0.59) |  | 3.50 (0.55) | 3.74 (0.58) |  | 3.73 (0.60) | 3.37 (0.72) | 3.27 (0.77) | 3.74 (0.53) | 2.97 (0.83) |
| Indonesia |  | 3.58 (1.06) |  | 3.17 (1.19) |  | 3.37 (0.68) | 3.63 (0.61) |  | 3.42 (0.54) | 3.62 (0.59) |  | 3.41 (0.49) | 3.57 (0.58) |  | 3.55 (0.61) | 3.27 (0.70) | 3.25 (0.68) | 3.69 (0.53) | 3.01 (0.78) |
| Iran |  | 3.53 (1.17) |  | 2.45 (1.27) |  | 3.20 (0.72) | 3.71 (0.62) |  | 3.37 (0.60) | 3.67 (0.62) |  | 3.32 (0.55) | 3.63 (0.61) |  | 3.66 (0.63) | 3.41 (0.69) | 3.07 (0.74) | 3.72 (0.59) | 3.09 (0.84) |
| Ireland |  | 3.10 (1.20) |  | 2.21 (1.22) |  | 3.38 (0.74) | 3.68 (0.62) |  | 3.39 (0.61) | 3.67 (0.62) |  | 3.36 (0.56) | 3.76 (0.63) |  | 3.67 (0.66) | 3.38 (0.75) | 3.31 (0.79) | 3.65 (0.67) | 2.96 (0.82) |
| Israel |  | 3.29 (1.21) |  | 2.00 (1.30) |  | 3.31 (0.76) | 3.70 (0.63) |  | 3.45 (0.64) | 3.65 (0.64) |  | 3.48 (0.53) | 3.71 (0.64) |  | 3.61 (0.67) | 3.48 (0.76) | 3.18 (0.78) | 3.85 (0.56) | 2.97 (0.88) |
| Italy |  | 3.33 (1.18) |  | 2.26 (1.29) |  | 3.40 (0.72) | 3.65 (0.61) |  | 3.51 (0.60) | 3.60 (0.61) |  | 3.45 (0.53) | 3.62 (0.61) |  | 3.55 (0.63) | 3.46 (0.73) | 3.25 (0.76) | 3.86 (0.61) | 2.94 (0.82) |
| Jamaica |  | 3.61 (1.25) |  | 3.44 (1.32) |  | 3.30 (0.75) | 3.82 (0.64) |  | 3.44 (0.63) | 3.80 (0.64) |  | 3.37 (0.57) | 3.81 (0.63) |  | 3.76 (0.66) | 3.66 (0.77) | 3.16 (0.81) | 3.74 (0.60) | 2.95 (0.82) |
| Japan |  | 3.30 (1.18) |  | 2.24 (1.30) |  | 3.29 (0.76) | 3.60 (0.65) |  | 3.38 (0.60) | 3.60 (0.64) |  | 3.40 (0.55) | 3.64 (0.66) |  | 3.55 (0.69) | 3.36 (0.72) | 3.17 (0.80) | 3.74 (0.64) | 2.95 (0.08) |
| Jordan |  | 3.74 (1.16) |  | 3.05 (1.27) |  | 3.40 (0.73) | 3.75 (0.63) |  | 3.57 (0.59) | 3.67 (0.63) |  | 3.41 (0.53) | 3.74 (0.64) |  | 3.67 (0.66) | 3.56 (0.74) | 3.26 (0.73) | 3.77 (0.58) | 3.10 (0.86) |
| Kenya |  | 3.71 (1.17) |  | 3.50 (1.26) |  | 3.44 (0.73) | 3.88 (0.63) |  | 3.59 (0.61) | 3.82 (0.61) |  | 3.51 (0.57) | 3.86 (0.63) |  | 3.74 (0.65) | 3.73 (0.74) | 3.29 (0.79) | 3.83 (0.58) | 2.73 (0.79) |
| Kuwait |  | 3.64 (1.18) |  | 3.08 (1.25) |  | 3.28 (0.75) | 3.87 (0.61) |  | 3.45 (0.60) | 3.80 (0.6) |  | 3.38 (0.55) | 3.85 (0.59) |  | 3.81 (0.61) | 3.47 (0.72) | 3.13 (0.75) | 3.75 (0.52) | 3.10 (0.85) |
| Latvia |  | 3.43 (1.09) |  | 2.01 (1.19) |  | 3.28 (0.75) | 3.57 (0.58) |  | 3.47 (0.56) | 3.49 (0.62) |  | 3.41 (0.50) | 3.50 (0.63) |  | 3.50 (0.63) | 3.28 (0.69) | 3.15 (0.79) | 3.78 (0.50) | 2.94 (0.76) |
| Lebanon |  | 3.75 (1.09) |  | 2.93 (1.30) |  | 3.45 (0.65) | 3.72 (0.59) |  | 3.61 (0.55) | 3.61 (0.59) |  | 3.47 (0.50) | 3.72 (0.59) |  | 3.65 (0.61) | 3.52 (0.68) | 3.32 (0.68) | 3.81 (0.54) | 3.16 (0.81) |
| Lithuania |  | 3.43 (1.15) |  | 1.98 (1.14) |  | 3.23 (0.74) | 3.48 (0.62) |  | 3.39 (0.59) | 3.42 (0.60) |  | 3.37 (0.52) | 3.43 (0.63) |  | 3.39 (0.63) | 3.29 (0.69) | 3.11 (0.76) | 3.75 (0.53) | 3.10 (0.80) |
| Luxembourg |  | 3.13 (1.21) |  | 1.87 (1.21) |  | 3.35 (0.78) | 3.54 (0.61) |  | 3.29 (0.63) | 3.52 (0.63) |  | 3.46 (0.54) | 3.49 (0.63) |  | 3.43 (0.65) | 3.34 (0.78) | 3.25 (0.84) | 3.76 (0.62) | 3.15 (0.86) |
| Malaysia |  | 3.45 (1.11) |  | 3.02 (1.20) |  | 3.25 (0.68) | 3.69 (0.63) |  | 3.33 (0.55) | 3.66 (0.61) |  | 3.31 (0.53) | 3.61 (0.60) |  | 3.62 (0.62) | 3.18 (0.69) | 3.16 (0.70) | 3.56 (0.54) | 3.06 (0.77) |
| Malta |  | 3.13 (1.23) |  | 2.36 (1.26) |  | 3.36 (0.75) | 3.73 (0.61) |  | 3.42 (0.65) | 3.70 (0.60) |  | 3.40 (0.58) | 3.73 (0.62) |  | 3.68 (0.63) | 3.36 (0.71) | 3.26 (0.80) | 3.76 (0.67) | 3.12 (0.83) |
| Mauritius |  | 3.54 (1.21) |  | 3.06 (1.28) |  | 3.42 (0.76) | 3.69 (0.63) |  | 3.57 (0.67) | 3.66 (0.63) |  | 3.45 (0.62) | 3.64 (0.63) |  | 3.62 (0.65) | 3.42 (0.77) | 3.30 (0.82) | 3.76 (0.63) | 3.04 (0.87) |
| Mexico |  | 3.48 (1.24) |  | 2.53 (1.30) |  | 3.33 (0.78) | 3.62 (0.66) |  | 3.56 (0.67) | 3.53 (0.63) |  | 3.43 (0.59) | 3.53 (0.66) |  | 3.42 (0.66) | 3.36 (0.73) | 3.16 (0.81) | 3.87 (0.64) | 3.10 (0.85) |
| Morocco |  | 3.48 (1.17) |  | 2.99 (1.29) |  | 3.28 (0.72) | 3.69 (0.62) |  | 3.49 (0.58) | 3.59 (0.61) |  | 3.33 (0.58) | 3.64 (0.62) |  | 3.60 (0.65) | 3.36 (0.69) | 3.14 (0.75) | 3.79 (0.65) | 3.09 (0.80) |
| Netherlands |  | 3.14 (1.09) |  | 1.93 (1.24) |  | 3.57 (0.70) | 3.71 (0.55) |  | 3.52 (0.60) | 3.65 (0.57) |  | 3.49 (0.53) | 3.79 (0.57) |  | 3.66 (0.60) | 3.48 (0.69) | 3.51 (0.74) | 3.58 (0.64) | 2.80 (0.81) |
| Netherlands Antilles |  | 3.59 (1.15) |  | 2.67 (1.40) |  | 3.63 (0.71) | 3.74 (0.58) |  | 3.58 (0.61) | 3.65 (0.59) |  | 3.56 (0.55) | 3.77 (0.6) |  | 3.63 (0.61) | 3.65 (0.71) | 3.54 (0.75) | 3.71 (0.61) | 2.86 (0.77) |
| New Zealand |  | 3.26 (1.16) |  | 2.12 (1.34) |  | 3.38 (0.76) | 3.67 (0.62) |  | 3.42 (0.60) | 3.67 (0.62) |  | 3.42 (0.55) | 3.72 (0.63) |  | 3.64 (0.65) | 3.43 (0.73) | 3.29 (0.81) | 3.72 (0.62) | 2.91 (0.81) |
| Nicaragua |  | 3.66 (1.19) |  | 3.04 (1.32) |  | 3.37 (0.80) | 3.72 (0.63) |  | 3.61 (0.66) | 3.63 (0.61) |  | 3.43 (0.58) | 3.62 (0.63) |  | 3.50 (0.62) | 3.50 (0.71) | 3.16 (0.82) | 3.88 (0.62) | 3.05 (0.83) |
| Nigeria |  | 3.85 (1.06) |  | 3.70 (1.15) |  | 3.28 (0.64) | 3.90 (0.63) |  | 3.60 (0.57) | 3.84 (0.62) |  | 3.41 (0.53) | 3.84 (0.62) |  | 3.78 (0.64) | 3.62 (0.73) | 3.11 (0.68) | 3.77 (0.54) | 2.76 (0.77) |
| Norway |  | 3.23 (1.14) |  | 1.69 (1.08) |  | 3.34 (0.69) | 3.68 (0.58) |  | 3.42 (0.54) | 3.71 (0.59) |  | 3.44 (0.52) | 3.75 (0.59) |  | 3.68 (0.61) | 3.47 (0.67) | 3.28 (0.74) | 3.71 (0.61) | 2.77 (0.80) |
| Pakistan |  | 3.81 (1.11) |  | 3.33 (1.08) |  | 3.32 (0.71) | 3.81 (0.62) |  | 3.32 (0.59) | 3.74 (0.61) |  | 3.43 (0.57) | 3.75 (0.60) |  | 3.75 (0.60) | 3.38 (0.71) | 3.18 (0.75) | 3.66 (0.54) | 3.15 (0.84) |
| Panama |  | 3.76 (1.16) |  | 3.13 (1.29) |  | 3.50 (0.80) | 3.79 (0.61) |  | 3.67 (0.65) | 3.70 (0.60) |  | 3.54 (0.56) | 3.70 (0.61) |  | 3.57 (0.62) | 3.53 (0.72) | 3.28 (0.82) | 3.97 (0.59) | 2.98 (0.81) |
| Paraguay |  | 3.37 (1.22) |  | 2.97 (1.32) |  | 3.34 (0.79) | 3.63 (0.63) |  | 3.48 (0.63) | 3.56 (0.61) |  | 3.38 (0.56) | 3.55 (0.62) |  | 3.42 (0.63) | 3.38 (0.69) | 3.15 (0.80) | 3.86 (0.63) | 3.20 (0.84) |
| Peru |  | 3.53 (1.18) |  | 2.86 (1.25) |  | 3.42 (0.78) | 3.67 (0.60) |  | 3.58 (0.64) | 3.62 (0.59) |  | 3.46 (0.56) | 3.62 (0.61) |  | 3.48 (0.60) | 3.42 (0.70) | 3.22 (0.79) | 3.91 (0.58) | 3.04 (0.79) |
| Philippines |  | 3.43 (1.08) |  | 3.28 (1.15) |  | 3.32 (0.67) | 3.74 (0.61) |  | 3.38 (0.54) | 3.71 (0.60) |  | 3.39 (0.49) | 3.68 (0.59) |  | 3.65 (0.61) | 3.29 (0.67) | 3.16 (0.68) | 3.75 (0.52) | 3.08 (0.73) |
| Poland |  | 3.25 (1.23) |  | 2.18 (1.30) |  | 3.20 (0.79) | 3.46 (0.65) |  | 3.42 (0.62) | 3.39 (0.65) |  | 3.39 (0.54) | 3.41 (0.66) |  | 3.38 (0.69) | 3.15 (0.71) | 3.05 (0.82) | 3.84 (0.53) | 3.11 (0.83) |
| Portugal |  | 3.02 (1.23) |  | 1.98 (1.20) |  | 3.26 (0.73) | 3.65 (0.60) |  | 3.45 (0.58) | 3.63 (0.58) |  | 3.39 (0.55) | 3.62 (0.61) |  | 3.59 (0.62) | 3.25 (0.70) | 3.08 (0.78) | 3.91 (0.59) | 3.11 (0.82) |
| Puerto Rico |  | 3.80 (1.19) |  | 3.07 (1.33) |  | 3.58 (0.80) | 3.98 (0.60) |  | 3.68 (0.65) | 3.87 (0.59) |  | 3.53 (0.57) | 3.92 (0.61) |  | 3.80 (0.62) | 3.67 (0.71) | 3.35 (0.81) | 3.93 (0.63) | 2.93 (0.81) |
| Qatar |  | 3.52 (1.21) |  | 3.10 (1.25) |  | 3.36 (0.75) | 3.81 (0.63) |  | 3.46 (0.63) | 3.76 (0.63) |  | 3.42 (0.57) | 3.77 (0.62) |  | 3.73 (0.64) | 3.37 (0.72) | 3.22 (0.78) | 3.74 (0.57) | 3.11 (0.88) |
| Romania |  | 3.33 (1.24) |  | 2.50 (1.29) |  | 3.33 (0.77) | 3.55 (0.65) |  | 3.50 (0.61) | 3.50 (0.62) |  | 3.51 (0.58) | 3.48 (0.65) |  | 3.48 (0.66) | 3.29 (0.72) | 3.18 (0.81) | 3.95 (0.57) | 3.05 (0.82) |
| Russia |  | 3.51 (1.14) |  | 2.28 (1.24) |  | 3.32 (0.74) | 3.51 (0.63) |  | 3.50 (0.59) | 3.44 (0.62) |  | 3.40 (0.54) | 3.46 (0.65) |  | 3.41 (0.66) | 3.36 (0.71) | 3.2 (0.75) | 3.77 (0.56) | 3.03 (0.78) |
| Saudi Arabia |  | 3.65 (1.21) |  | 3.17 (1.16) |  | 3.31 (0.71) | 3.85 (0.61) |  | 3.50 (0.60) | 3.77 (0.60) |  | 3.40 (0.55) | 3.80 (0.58) |  | 3.77 (0.60) | 3.43 (0.73) | 3.17 (0.72) | 3.75 (0.58) | 3.07 (0.84) |
| Serbia-Montenegro |  | 3.41 (1.20) |  | 2.30 (1.31) |  | 3.40 (0.73) | 3.59 (0.60) |  | 3.50 (0.57) | 3.52 (0.59) |  | 3.56 (0.52) | 3.54 (0.61) |  | 3.52 (0.63) | 3.31 (0.71) | 3.26 (0.77) | 3.91 (0.51) | 3.08 (0.85) |
| Singapore |  | 3.28 (1.14) |  | 2.62 (1.23) |  | 3.21 (0.72) | 3.58 (0.62) |  | 3.31 (0.58) | 3.57 (0.61) |  | 3.26 (0.55) | 3.53 (0.61) |  | 3.52 (0.63) | 3.10 (0.69) | 3.13 (0.76) | 3.57 (0.58) | 3.10 (0.77) |
| Slovak Republic |  | 3.17 (1.24) |  | 2.27 (1.35) |  | 3.40 (0.82) | 3.62 (0.61) |  | 3.50 (0.66) | 3.55 (0.62) |  | 3.51 (0.59) | 3.59 (0.64) |  | 3.54 (0.65) | 3.37 (0.75) | 3.26 (0.85) | 3.83 (0.56) | 2.91 (0.92) |
| Slovenia |  | 3.22 (1.25) |  | 1.84 (1.20) |  | 3.34 (0.79) | 3.52 (0.66) |  | 3.49 (0.61) | 3.44 (0.64) |  | 3.49 (0.58) | 3.46 (0.69) |  | 3.43 (0.70) | 3.29 (0.75) | 3.22 (0.84) | 3.86 (0.59) | 2.98 (0.82) |
| South Africa |  | 3.32 (1.20) |  | 2.97 (1.40) |  | 3.37 (0.76) | 3.70 (0.63) |  | 3.48 (0.61) | 3.67 (0.62) |  | 3.46 (0.56) | 3.71 (0.63) |  | 3.62 (0.66) | 3.52 (0.72) | 3.26 (0.81) | 3.79 (0.60) | 2.98 (0.81) |
| South Korea |  | 3.56 (1.08) |  | 2.42 (1.33) |  | 3.26 (0.70) | 3.66 (0.58) |  | 3.34 (0.57) | 3.66 (0.57) |  | 3.37 (0.51) | 3.67 (0.58) |  | 3.61 (0.60) | 3.44 (0.66) | 3.16 (0.74) | 3.65 (0.61) | 2.89 (0.71) |
| Spain |  | 3.08 (1.27) |  | 1.90 (1.16) |  | 3.42 (0.78) | 3.75 (0.59) |  | 3.48 (0.64) | 3.65 (0.59) |  | 3.37 (0.57) | 3.70 (0.6) |  | 3.59 (0.61) | 3.26 (0.73) | 3.29 (0.82) | 3.76 (0.64) | 3.11 (0.85) |
| Sri Lanka |  | 3.64 (1.09) |  | 3.34 (1.28) |  | 3.39 (0.67) | 3.77 (0.56) |  | 3.48 (0.58) | 3.75 (0.55) |  | 3.46 (0.50) | 3.76 (0.55) |  | 3.72 (0.56) | 3.43 (0.68) | 3.23 (0.72) | 3.80 (0.53) | 3.02 (0.79) |
| Suriname |  | 3.80 (1.08) |  | 3.18 (1.29) |  | 3.54 (0.68) | 3.78 (0.61) |  | 3.53 (0.56) | 3.64 (0.61) |  | 3.56 (0.59) | 3.76 (0.59) |  | 3.62 (0.60) | 3.71 (0.68) | 3.45 (0.72) | 3.75 (0.58) | 2.84 (0.75) |
| Sweden |  | 3.35 (1.14) |  | 1.67 (1.10) |  | 3.38 (0.73) | 3.64 (0.61) |  | 3.46 (0.56) | 3.65 (0.62) |  | 3.49 (0.52) | 3.70 (0.62) |  | 3.62 (0.65) | 3.43 (0.71) | 3.30 (0.78) | 3.77 (0.60) | 2.81 (0.80) |
| Switzerland |  | 3.43 (1.07) |  | 2.05 (1.26) |  | 3.61 (0.71) | 3.74 (0.55) |  | 3.47 (0.59) | 3.76 (0.57) |  | 3.60 (0.51) | 3.71 (0.56) |  | 3.63 (0.59) | 3.64 (0.73) | 3.54 (0.77) | 3.74 (0.63) | 2.76 (0.80) |
| Taiwan |  | 3.55 (1.10) |  | 2.54 (1.30) |  | 3.22 (0.71) | 3.60 (0.58) |  | 3.27 (0.59) | 3.62 (0.57) |  | 3.33 (0.54) | 3.62 (0.60) |  | 3.57 (0.59) | 3.34 (0.67) | 3.12 (0.74) | 3.59 (0.61) | 2.99 (0.76) |
| Thailand |  | 3.52 (1.07) |  | 2.74 (1.27) |  | 3.35 (0.70) | 3.71 (0.61) |  | 3.40 (0.57) | 3.68 (0.60) |  | 3.44 (0.52) | 3.68 (0.59) |  | 3.65 (0.61) | 3.43 (0.67) | 3.23 (0.71) | 3.64 (0.59) | 2.89 (0.75) |
| Trinidad and Tobago |  | 3.41 (1.24) |  | 3.08 (1.29) |  | 3.29 (0.76) | 3.73 (0.65) |  | 3.47 (0.61) | 3.67 (0.64) |  | 3.37 (0.58) | 3.71 (0.64) |  | 3.65 (0.66) | 3.48 (0.79) | 3.14 (0.81) | 3.80 (0.60) | 2.98 (0.83) |
| Turkey |  | 3.64 (1.13) |  | 2.39 (1.32) |  | 3.44 (0.74) | 3.66 (0.62) |  | 3.51 (0.61) | 3.66 (0.61) |  | 3.53 (0.53) | 3.66 (0.60) |  | 3.61 (0.63) | 3.48 (0.73) | 3.30 (0.76) | 3.84 (0.54) | 2.99 (0.80) |
| United Arab Emirates |  | 3.64 (1.15) |  | 2.97 (1.30) |  | 3.43 (0.71) | 3.79 (0.61) |  | 3.51 (0.60) | 3.75 (0.61) |  | 3.46 (0.55) | 3.78 (0.60) |  | 3.72 (0.62) | 3.47 (0.73) | 3.31 (0.74) | 3.75 (0.57) | 2.97 (0.83) |
| United Kingdom |  | 3.01 (1.22) |  | 1.90 (1.22) |  | 3.38 (0.77) | 3.60 (0.66) |  | 3.42 (0.62) | 3.59 (0.65) |  | 3.40 (0.56) | 3.66 (0.67) |  | 3.58 (0.69) | 3.32 (0.75) | 3.29 (0.82) | 3.72 (0.63) | 3.01 (0.84) |
| United States |  | 3.41 (1.20) |  | 2.70 (1.43) |  | 3.42 (0.80) | 3.76 (0.66) |  | 3.43 (0.62) | 3.75 (0.65) |  | 3.42 (0.57) | 3.82 (0.67) |  | 3.71 (0.69) | 3.52 (0.73) | 3.31 (0.85) | 3.72 (0.66) | 2.92 (0.83) |
| Uruguay |  | 3.19 (1.26) |  | 2.19 (1.28) |  | 3.40 (0.78) | 3.61 (0.60) |  | 3.51 (0.63) | 3.55 (0.60) |  | 3.38 (0.56) | 3.59 (0.61) |  | 3.43 (0.62) | 3.29 (0.72) | 3.24 (0.80) | 3.82 (0.64) | 3.23 (0.81) |
| Venezuela |  | 3.66 (1.19) |  | 2.93 (1.30) |  | 3.48 (0.80) | 3.74 (0.63) |  | 3.64 (0.65) | 3.63 (0.61) |  | 3.50 (0.56) | 3.65 (0.63) |  | 3.52 (0.63) | 3.44 (0.70) | 3.26 (0.83) | 3.96 (0.61) | 3.07 (0.82) |
| Vietnam |  | 3.73 (1.08) |  | 2.48 (1.34) |  | 3.37 (0.69) | 3.75 (0.58) |  | 3.48 (0.56) | 3.72 (0.58) |  | 3.41 (0.52) | 3.71 (0.58) |  | 3.66 (0.60) | 3.49 (0.69) | 3.22 (0.72) | 3.68 (0.56) | 2.86 (0.74) |

Note: TS = target scale, AC = ant colony, BF = brute force, agy = agency, com = communion, agr = agreeableness, cns = conscientiousness, ext = extraversion, opn = openness, neu = neuroticism.

**Section S4.** Table S7 reports on the properties of our Big Two and Big Five scales: number of items, mean internal consistency across countries, and measurement invariance (MI) across countries. The table shows that Cronbach’s αs of two Big Two Scales (AC and BF) were rather low (.52 ≤ αs ≤ .68). However, those Big Two Scales assess rather broad constructs with rather specific items. In such cases, Cronbach’s α largely indicates construct breadth, rather than amount of true-score variance (Schmitt, 1996). To illustrate, the GSOEP version of the Big Five Inventory is another measure that assesses broad traits (the Big Five) with few, rather narrow items (3 items per Big Five trait). As a result, that inventory has modest αs (.44 < αs < .75), even though the inventory’s reliability is acceptable (as indicated by retest correlations of *r*s > .75; Lang, 2005). Moreover, the results of those two Big Two Scales (AC and BF) were conceptually identical to the results of the third Big Two Scale (TS) and the latter had satisfactory αs (≥ .70). Finally, the results of all three Big Two Scales were theory-consistent (Gebauer et al., 2012, 2013, 2014).

The tests for MI also warrant some explanation. There exist different levels of MI, varying in their strictness. A comparatively weak form of MI (Meredith, 1993) is necessary and sufficient for our research question (i.e., are there cross-cultural differences in the degree to which two measures are related to each other?) (Horn & McArdle, 1992). That form is typically called *metric* MI and its calculation comprises three steps (Raju et al., 2002): (1) One computes a configural model. A configural model is a multi-group CFA model with a latent variable (e.g., agency) defined by a set of items (here: e.g., the 8 items of the TS-Agency Scale) and no constraints across groups (here: all scale properties can vary freely across countries). Our models also included a latent method variable defined by all reverse-keyed items (if there were any; Geiser & Lockhart, 2012). (2) One also computes a metric model, which differs from the configural model in one key respect (otherwise it is identical). Specifically, the loading of each item (here: the 8 TS-Agency items) on its latent variable (here: agency) is set to be equal across groups (here: countries). (3) One inspects the absolute fit of the two models (configural and metric) and calculates their difference (∆fit). The metric model’s fit is typically worse than the configural model’s fit because the former has more restrictions across groups. One grants the scale metric MI if the two models’ absolute fits are acceptable and if their ∆fit lies within an acceptable threshold (extant thresholds are: ∆fit ≤ .05—Little, 1997; ∆fit ≤ .022—McGaw & Jöreskog, 1971; ∆fit ≤ .01—Cheung & Rensvold, 2002).

Table S7 shows that the ER-Big Two Scales’ absolute fit were unacceptably low. For that reason, we do not further report on those scales in the present paper. Importantly, however, the table also shows that all other scales possess much more acceptable levels of metric MI. In particular, we considered three fit indices (comparative fit index [*CFI*], root mean square error of approximation [*RMSEA*], and standardized root mean square residual [*SRMR*]) and found that our scales met the most conservative threshold (∆fit ≤ .01) in 28 out of 33 cases (85%). The more moderate threshold (∆fit ≤ .022) was met in another four cases (12%) and that threshold was surpassed in a single case only (Neuroticism Scale: ∆*SRMR* = .03). Notably, though, the TS-Agency Scale had a high *RMSEA* (.11) and the AC-Agency Scale had a somewhat low *CFI* in the metric model (.89). Yet, the substantive results of the TS-Agency and the AC-Agency Scales were highly consistent with the results of the BF-Agency Scale and the latter evidenced acceptable model fit/measurement invariance. Moreover, the results of the TS-Agency and the AC-Agency Scales were highly theory-consistent. Finally, the TS-Agency and the AC-Agency Scales evidenced acceptable model fit/measurement invariance in the U.S. state-level analyses in Study S2 (see footnote 2) and Study S3 (see footnote 3). For all those reasons it seems unlikely that our results are spuriously caused by issues with MI. On the contrary, those issues most likely increased error variance and, thus, if anything, worked against our hypotheses.^S6^

To contextualize our MI results, a general note about MI appears helpful. How should one interpret cross-cultural results if MI is imperfect (and it typically is)? The standard scholarly response is “with caution.” That response is based on the assumption that imperfect MI provides an *alternative explanation* to the uncovered results. Yet, imperfect MI provides a viable alternative explanation only for a specific type of research and our research is not of that type. More precisely, we predicted a very specific result-pattern across 102 countries and found strong support for it. It is extremely unlikely that imperfect MI (i.e., some differences in the meaning of our constructs across our 102 countries) happens to result in precisely the result pattern that we predicted across those 102 countries on the basis of *a priori* theory (Gebauer et al., 2013, 2014). On the contrary, it appears much more justifiable to consider imperfect MI as a form of measurement error which works against our predictions. From that point of view, it speaks to the validity of our theoretical predictions that we found strong empirical support for them even though MI was (unsurprisingly) imperfect. Stated more generally, the probability that imperfect MI offers an alternative explanation to a given result pattern decreases with increasing theoretical clarity of the predictions and with increasing numbers of groups involved in those predictions (here: 102 countries). This notion is underappreciated in cross-cultural psychology and also in other psychological subfields concerned with MI.

**Table S7.** Properties of the Big Two and Big Five Scales (Number of Items, Mean Internal Consistency Across Countries, and Measurement Invariance Across Countries)

|  |  | **Big Two** | | | | | | | | | | |  | **Big Five** | | | | |
| --- | --- | --- | --- | --- | --- | --- | --- | --- | --- | --- | --- | --- | --- | --- | --- | --- | --- | --- |
|  |  | **ER** | |  | **TS** | |  | **AC** | |  | **BF** | |  |  |  |  |  |  |
|  |  | **agy** | **com** |  | **agy** | **com** |  | **agy** | **com** |  | **agy** | **com** |  | **agr** | **cns** | **opn** | **ext** | **neu** |
| number of items |  | 8 | 9 |  | 8 | 8 |  | 6 | 7 |  | 8 | 8 |  | 9 | 9 | 10 | 8 | 8 |
|  |  |  |  |  |  |  |  |  |  |  |  |  |  |  |  |  |  |  |
| Cronbach's α | (*M*/*SD*) | .78/.02 | .70/.04 |  | .81/.02 | .70/.03 |  | .52/.05 | .62/.05 |  | .53/.04 | .68/.04 |  | .73/.04 | .82/.02 | .74/.04 | .82/.03 | .83/.02 |
|  |  |  |  |  |  |  |  |  |  |  |  |  |  |  |  |  |  |  |
| MI | model |  |  |  |  |  |  |  |  |  |  |  |  |  |  |  |  |  |
|  |  |  |  |  |  |  |  |  |  |  |  |  |  |  |  |  |  |  |
| CFI | config | .76 | .65 |  | .91 | .96 |  | .91 | .97 |  | .93 | .94 |  | .94 | .98 | .84 | .94 | .95 |
|  | metric | .74 | .63 |  | .90 | .95 |  | .89 | .96 |  | .91 | .93 |  | .93 | .97 | .83 | .93 | .93 |
|  | Δ | .02 | .02 |  | .01 | .01 |  | .02 | .01 |  | .02 | .01 |  | .01 | .01 | .01 | .01 | .02 |
|  |  |  |  |  |  |  |  |  |  |  |  |  |  |  |  |  |  |  |
| RMSEA | config | .17 | .16 |  | .11 | .05 |  | .07 | .05 |  | .07 | .07 |  | .06 | .05 | .11 | .10 | .08 |
|  | metric | .15 | .14 |  | .10 | .05 |  | .06 | .05 |  | .07 | .06 |  | .06 | .05 | .10 | .09 | .08 |
|  | Δ | .02 | .02 |  | .01 | .00 |  | .01 | .00 |  | .00 | .01 |  | .00 | .00 | .01 | .01 | .00 |
|  |  |  |  |  |  |  |  |  |  |  |  |  |  |  |  |  |  |  |
| SRMR | config | .09 | .10 |  | .05 | .03 |  | .03 | .02 |  | .05 | .03 |  | .03 | .02 | .06 | .04 | .03 |
|  | metric | .10 | .11 |  | .06 | .04 |  | .04 | .03 |  | .05 | .04 |  | .04 | .03 | .07 | .06 | .06 |
|  | Δ | -.01 | -.01 |  | -.01 | -.01 |  | -.01 | -.01 |  | .00 | -.01 |  | -.01 | -.01 | -.01 | -.02 | -.03 |

Note: ER = expert rating, TS = target scale, AC = ant colony, BF = brute force; agy = agency, com = communion, agr = agreeableness, cns = conscientiousness, ext = extraversion, opn = openness, neu = neuroticism, CFI = comparative fit index, RMSEA = root mean square error of approximation, SRMR = standardized root mean square residual, config = configural model, metric = metric model Δ = fit difference between the two models.

**Section S5.** Here, we describe which BFI items belong to which BFI-Big Two Scales. For copyright reasons, we report the BFI-item *numbers*, rather than the items themselves (the items can be found in John & Srivastava, 1999): ER-Big Two Scale (agency: 1, 5, 10, 11, 16, 25, 26, 36; communion: 3, 7, 13, 17, 22, 28, 32, 38, 42), TS-Big Two Scale (agency: 1, 5, 11, 16, 21, 26, 31, 36; communion: 2, 7, 12, 17, 32, 33, 37, 42), AC-Big Two Scale (agency: 11, 20, 26, 31, 34, 35; communion: 2, 3, 12, 22, 32, 37, 42), BF-Big Two Scale (agency: 5, 6, 9, 15, 21, 25, 28, 44; communion: 3, 7, 12, 17, 27, 32, 37, 42).

**Supplementary References**

Andresen, E. M., Malmgren, J. A., Carter, W. B., & Patrick, D. L. (1994). Screening for depression in well older adults: Evaluation of a short form of the CES-D. *American Journal of Preventive Medicine*, *10*, 77-84.

Buhrmester, M., Kwang, T., & Gosling, S. D. (2011). Amazon's Mechanical Turk: A new source of inexpensive, yet high-quality, data?. *Perspectives on Psychological Science, 6*, 3-5.

Cheung, G. W., & Rensvold, R. B. (2002). Evaluating goodness-of-fit indexes for testing measurement invariance. *Structural Equation Modeling, 9,* 233-255.

Diener, E., Tay, L., & Myers, D. G. (2011). The religion paradox: If religion makes people happy, why are so many dropping out? *Journal of Personality and Social Psychology, 101,* 1278-1290.

Funder, D. C., & Colvin, C. R. (1988). Friends and strangers: Acquaintanceship, agreement, and the accuracy of personality judgment. *Journal of Personality and Social Psychology, 55,* 149-158.

Gallup (2018, December 21). State of the States. Retrieved from https://news.gallup.com/poll/125066/state-states.aspx

Gebauer, J. E., Bleidorn, W., Gosling, S. D., Rentfrow, P. J., Lamb, M. E., & Potter, J. (2014). Cross-cultural variations in Big Five relationships with religiosity: A sociocultural motives perspective. *Journal of Personality and Social Psychology, 107*, 1064-1091.

Gebauer, J. E., Leary, M. R., & Neberich, W. (2012). Big Two personality and Big Three mate preferences: Similarity attracts, but country-level mate preferences crucially matter. *Personality and Social Psychology Bulletin, 38,* 1579-1593.

Gebauer, J. E., Paulhus, D. L., & Neberich, W. (2013). Big two personality and religiosity across cultures: Communals as religious conformists and agentics as religious contrarians. *Social Psychological and Personality Science, 4*, 21-30.

Gebauer, J. E., Sedikides, C., Schönbrodt, F. D., Bleidorn, W., Rentfrow, P. J., Potter, J., & Gosling, S. D. (2017). The religiosity as social value hypothesis: A multi-method replication and extension across 65 countries and three levels of spatial aggregation. *Journal of Personality and Social Psychology*, *113*, e18-e39.

Gebauer, J. E., Sedikides, C., Wagner, J., Bleidorn, W., Rentfrow, P. J., Potter, J., & Gosling, S. D. (2015). Cultural norm fulfillment, interpersonal belonging, or getting ahead? A large-scale cross-cultural test of three perspectives on the function of self-esteem. *Journal of Personality and Social Psychology, 109,*526-548.

Geiser, C., & Lockhart, G. (2012). A comparison of four approaches to account for method effects in latent state-trait analyses. *Psychological Methods, 17,* 255-283.

Hogan, R., Hogan, J., & Roberts, B. W. (1996). Personality and employment decisions. *American Psychologist, 51,* 469-477.

Horn, J. L., & McArdle, J. J. (1992). A practical and theoretical guide to measurement invariance in aging research. *Experimental Aging Research, 18,* 117-144.

Inglehart, R., & Baker, W. E. (2000). Modernization, cultural change, and the persistence of traditional values. *American Sociological Review, 65*, 19-51.

John, O. P., Donahue, E. M., & Kentle, R. L. (1991). *The Big Five Inventory: Versions 4a and 54*. Berkeley, CA: University of California, Institute of Personality and Social Research.

John, O. P., & Srivastava, S. (1999). The Big Five trait taxonomy: History, measurement, and theoretical perspectives. In L. A. Pervin & O. P. John (Eds.), Handbook of personality: Theory and research (2nd ed., pp. 102-138). New York: Guilford Press.

Joshanloo, M., & Gebauer, J. E. (2020). Religiosity’s nomological network and temporal change: Introducing an extensive country-level religiosity index based on Gallup World Poll data. *European Psychologist, 25,* 26-40.

Kenny, D. A., & McCoach, D. B. (2003). Effect of the number of variables on measures of fit in Structural Equation Modeling. *Structural Equation Modeling, 10,* 333-351.

Lang, F. R. (2005). *Erfassung des kognitiven Leistungspotenzials und der “Big Five” mit Computer-Assisted-Personal-Interviewing (CAPI): Zur* *Reliabilität und Validität zweier ultrakurzer Tests und des BFI-SS* [Measuring the cognitive achievement potential and the Big Five with computer-assisted-personal-interviewing (CAPI): Reliability and validity of two short tests and the BFI]. Berlin, Germany: Deutsches Institut für Wirtschaftsforschung (DIW) Berlin.

Little, T. D. (1997). Mean and covariance structures (MACS) analyses of cross-cultural data: Practical and theoretical issues. *Multivariate Behavioral Research, 32,* 53-76.

McGaw, B., & Jöreskog, K. G. (1971). Factorial invariance of ability measures in groups differing in intelligence and socio-economic status. *The British Journal of Mathematical and Statistical Psychology, 24,* 154-168.

Meade, A. W., & Craig, S. B. (2012). Identifying careless responses in survey data. *Psychological Methods, 17*, 437-455.

Meredith, W. (1993). Measurement invariance, factor analysis, and factorial invariance. *Psychometrika, 58,* 525-543.

Paulhus, D. L., & Vazire, S. (2007). The self-report method. In R. W. Robins, R. C. Fraley, & R. Krueger (Eds.), *Handbook of research methods in personality psychology* (pp. 224–239). New York, NY: Guilford Press.

Pyszczynski, T., Greenberg, J., Solomon, S., Arndt, J., Schimel, J. (2004). Why do people need self-esteem? A theoretical and empirical review. *Psychological Bulletin, 130*, 435-468.

Raju, N. S., Laffitte, L. J., & Byrne, B. M. (2002). Measurement equivalence: A comparison of methods based on confirmatory factor analysis and item response theory. *Journal of Applied Psychology, 87,* 517-529.

Rentfrow, P. J., Gosling, S. D., & Potter, J. (2008). A theory of the emergence, persistence, and expression of geographic variation in psychological characteristics. *Perspectives on Psychological Science*, *3*, 339-369.

Rosenberg, M. (1965). *Society and the adolescent self-image.* Princeton, NJ: Princeton University Press.

Schmitt, N. (1996). Uses and abuses of coefficient alpha. *Psychological Assessment*, *8*, 350-353.

Soto, C. J., & John, O. P. (2009). Ten facet scales for the Big Five Inventory: Convergence with NEO PI-R facets, self-peer agreement, and discriminant validity. *Journal of Research in Personality, 43*, 84-90.

Soto, C. J., & John, O. P. (2017). The next Big Five Inventory (BFI-2): Developing and assessing a hierarchical model with 15 facets to enhance bandwidth, fidelity, and predictive power. *Journal of Personality and Social Psychology,* *113*, 117-143.

Stavrova, O., Fetchenhauer, D., & Schlösser, T. (2013). Why are religious people happy? The effect of the social norm of religiosity across countries. *Social Science Research*, *42*, 90-105.

Talhelm, T., Zhang, X., Oishi, S., Shimin, C., Duan, D., Lan, X., & Kitayama, S. (2014). Large-scale psychological differences within China explained by rice versus wheat agriculture. *Science, 344*, 603-608.

Vazire, S. (2010). Who knows what about a person? The self-other knowledge asymmetry (SOKA) model. *Journal of Personality and Social Psychology, 98,* 281-300.

Vazire, S., & Mehl, M. R. (2008). Knowing me, knowing you: The accuracy and unique predictive validity of self-ratings and other-ratings of daily behavior. *Journal of Personality and Social Psychology, 95,* 1202-1216.

Watson, D., Clark, L. A., & Tellegen, A. (1988). Development and validation of brief measures of positive and negative affect: the PANAS scales. *Journal of Personality and Social Psychology*, *54,* 1063-1070.

**Supplementary Notes**

S1. Section S4 describes measurement-invariance tests for the main-text data. That online supplement also describes some general guidelines on measurement-invariance testing. Among other things, it describes how recommendations differ regarding the threshold for acceptable measurement invariance. More precisely, Little (1997) recommended a rather lenient threshold (∆fit ≤ .05; see Section S4 for more information), McGaw and Jöreskog (1971) recommended a moderate threshold (∆fit ≤ .022), and Cheung and Rensvold (2002) recommended a conservative threshold (∆fit ≤ .01). For Study S1’s data, we received the following measurement-invariance results: Our scales met the most conservative threshold (∆fit ≤ .01) in 24 out of 33 cases (73%), they met the more moderate threshold (∆fit ≤ .022) in four additional cases (12%), and they met the lenient threshold in the five remaining cases (15%). Those results suggest sufficient measurement invariance.

S2. The present analyses revealed even more satisfactory measurement invariance than did the main-text analyses: Our scales met the most conservative threshold (∆fit ≤ .01) in all 33 cases. It is not surprising that our scales evidenced such a high level of measurement invariance, because the present analyses concern invariance across federal states within a single country, whereas the main-text analyses concern invariance across countries from all over the world.

S3. The present analyses revealed even more satisfactory measurement invariance than the main-text analyses: Our scales met the most conservative threshold (∆fit ≤ .01) in 32 out of 33 cases (97%) and they met the more moderate threshold (∆fit ≤ .022) in the remaining case (3%).

S4. This item belongs to the BFI’s Neuroticism Scale (John et al., 1991), but it is well-suited as a single-item scale of depression: We ran a study on Amazon’s Mechanical Turk (*N* = 511; 52.00% women, *M*_age_ = 35,37 years, *SD*_age_ = 1,52; Buhrmester et al., 2011) that included the BFI’s Neuroticism Scale together with the 10-item short form of the Center for Epidemiologic Studies Depression Scale (CES-D; Andresen et al., 1994). We instructed participants to complete the CES-D in a trait variant (i.e., “Please indicate how much the following 10 statements are typically true for you;” cf. Watson et al., 1988). The single-item depression scale evidenced a near-perfect (manifest) correlation with the CES-D, *r*(510) = .87, 95% CI [.85, .89]. We also considered measuring depression with Soto and John’s (2009) Depression subscale of the BFI’s Neuroticism Scale. That subscale contains two items: “I see myself as someone who is depressed, blue” (i.e., the single-item depression scale) and “I see myself as someone who can be moody.” We decided against using the two-item Depression subscale for three reasons: (1) The face/content validity of the “moody”-item appears low. (2) The correlation between the Depression subscale and the CES-D was descriptively lower than the correlation between the single-item depression scale and the CES-D, even though the Depression subscale includes twice as many items (*r* = .85 vs. *r* = .87). (3) Soto and John’s (2017) BFI-2 provides an update of the Depression subscale and they discarded the “moody”-item from their revised Depression subscale (instead, they placed a variant of the item in their newly added Emotional Volatility subscale).

S5. The single-item depression scale is an item from the BFI’s Neuroticism Scale. We needed to ensure that the item did not appear twice in the Big Five model (as criterion and as part of our neuroticism score). Therefore, for Study S4, we excluded the depression item from our neuroticism score.

S6. The absolute model fits of the Openness Scale were somewhat too low (Table S7). They improved considerably, however, when four item-parcels served as indicators of openness (configural model: *CFI* = .97, *RMSEA* = .13, *SRMR* = .03; metric model: *CFI* = .96, *RMSEA* = .10, *SRMR* = .04; cf. Kenny & McCoach, 2003).
